# Supplementary material for: Flexible Usage and Interconnectivity of Diverse Cell Death Pathways Protect against Intracellular Infection
Source: Immunity. 2020 Sep 15;53(3):533–547.e7. doi: 10.1016/j.immuni.2020.07.004 (PMC7500851; doi:10.1016/j.immuni.2020.07.004)
Supplement: Document S1. Figures S1–S6 [file mmc1.pdf]

Immunity, Volume *53*

Supplemental Information

Flexible Usage and Interconnectivity of Diverse Cell Death Pathways Protect against Intracellular Infection

Marcel Doerflinger, Yexuan Deng, Paul Whitney, Ranja Salvamoser, Sven Engel, Andrew J. Kueh, Lin Tai, Annabell Bachem, Elise Gressier, Niall D. Geoghegan, Stephen Wilcox, Kelly L. Rogers, Alexandra L. Garnham, Michael A. Dengler, Stefanie M. Bader, Gregor Ebert, Jaclyn S. Pearson, Dominic De Nardo, Nancy Wang, Chenying Yang, Milton Pereira, Clare E. Bryant, Richard A. Strugnell, James E. Vince, Marc Pellegrini, Andreas Strasser, Sammy Bedoui, and Marco J. Herold

SUPPLEMENTAL INFORMATION

Supplementary Figures

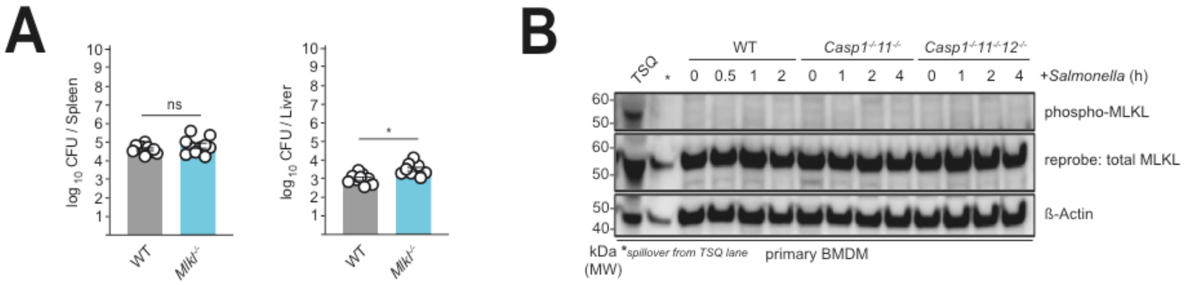

Figure S1 (related to Figure 1): Necroptosis Does not Affect Bacterial Clearance or Provide Back-up for Loss of Pyroptosis

(A) Bacterial loads in spleen and liver 3-week post infection in WT and *Mlkl*$^{-/-}$ mice infected with *Salmonella ΔAroA* (200 CFU). *N*=8 mice per group. Mean and SEM are shown. *$p<0.05$, $^{ns}p>0.05$=not significant.

(B) WT, *Casp1*$^{-/-}$*;Casp11*$^{-/-}$ and *Casp1*$^{-/-}$*;Casp11*$^{-/-}$*;Casp12*$^{-/-}$ BMDMs were infected with *Salmonella* SL1344 (MOI=50) and phosphorylation of MLKL analyzed by Western blotting at the indicated time points. Probing for ß-actin served as a loading control. Treatment with TNF-α+ Smac mimetic + caspase inhibitor QVD-OPH (TSQ) was used as a positive control for activation (i.e. phosphorylation) of MLKL and induction of necroptosis.

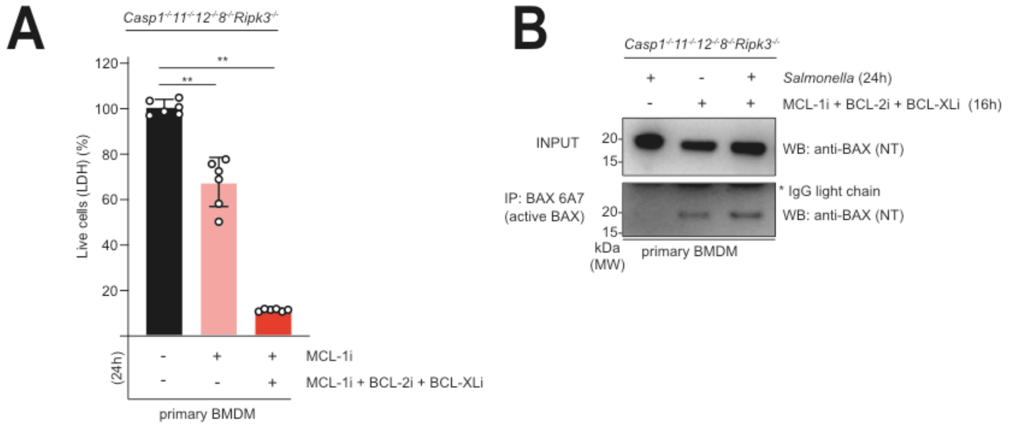

Figure S2 (related to Figure 2). Primary BMDMs from *Casp1–/–;Casp11–/–;Casp12–/–;Casp8–/–;Ripk3–/–* Mice Are Susceptible to Intrinsic Apoptosis Induced by BH3 Mimetic Drugs.

(A) *Casp1–/–;Casp11–/–;Casp12–/–;Casp8–/–;Ripk3–/–* BMDMs were treated with BH3 mimetic drugs individually (2 µM MCL1i) or in combination (each 2 µM of MCL-1i + BCL-2i + BCL-XLi) as indicated. Cell death was measured using an LDH release assay. Data pooled from 2 or more experiments. Mean and SD are shown. **P<0.005.

(B) Immunoprecipitation and immunoblotting of activated BAX in *Casp1–/–;Casp11–/–;Casp12–/–;Casp8–/–;Ripk3–/–* BMDMs infected with *Salmonella* SL1344 (MOI=50) and/or treated with the BH3 mimetic drug combination (each 2 µM of MCL-1i + BCL-2i + BCL-XLi).

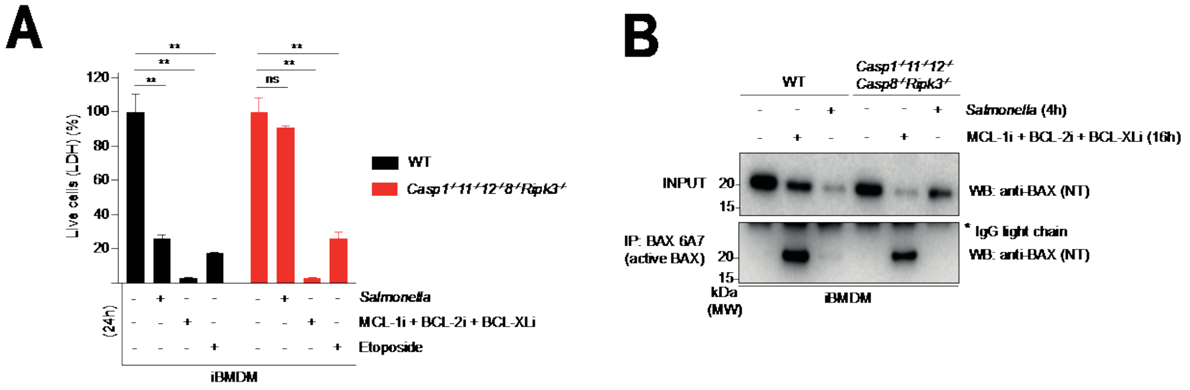

Figure S3 (related to Figure 3). ***Casp1***$^{-/-}$***;Casp11***$^{-/-}$***;Casp12***$^{-/-}$***;Casp8***$^{-/-}$***;Ripk3***$^{-/-}$ **iBMDMs Undergo Intrinsic Apoptosis in Response to BH3 Mimetic Drugs or Etoposide**

(A) LDH release cell death assay of WT and *Casp1*$^{-/-}$*;Casp11*$^{-/-}$*;Casp12*$^{-/-}$*;Casp8*$^{-/-}$*;Ripk3*$^{-/-}$ iBMDMs after 24 h treatment with a combination of BH3 mimetic drugs (each 2 µM of MCL-1i + BCL-2i + BCL-XLi), Etoposide (50 µM) or infection with *Salmonella* SL1344 (MOI=50). Mean and SEM are shown. **p<0.005; ^{ns}p>0.05=not significant.

(B) Immunoprecipitation and immunoblotting of activated BAX in WT and *Casp1*$^{-/-}$*;Casp11*$^{-/-}$*;Casp12*$^{-/-}$*;Casp8*$^{-/-}$*;Ripk3*$^{-/-}$ iBMDMs infected with *Salmonella* SL1344 (MOI=50) or treated with a BH3 mimetic drug combination (each 2 µM of MCL-1i + BCL-2i + BCL-XLi).

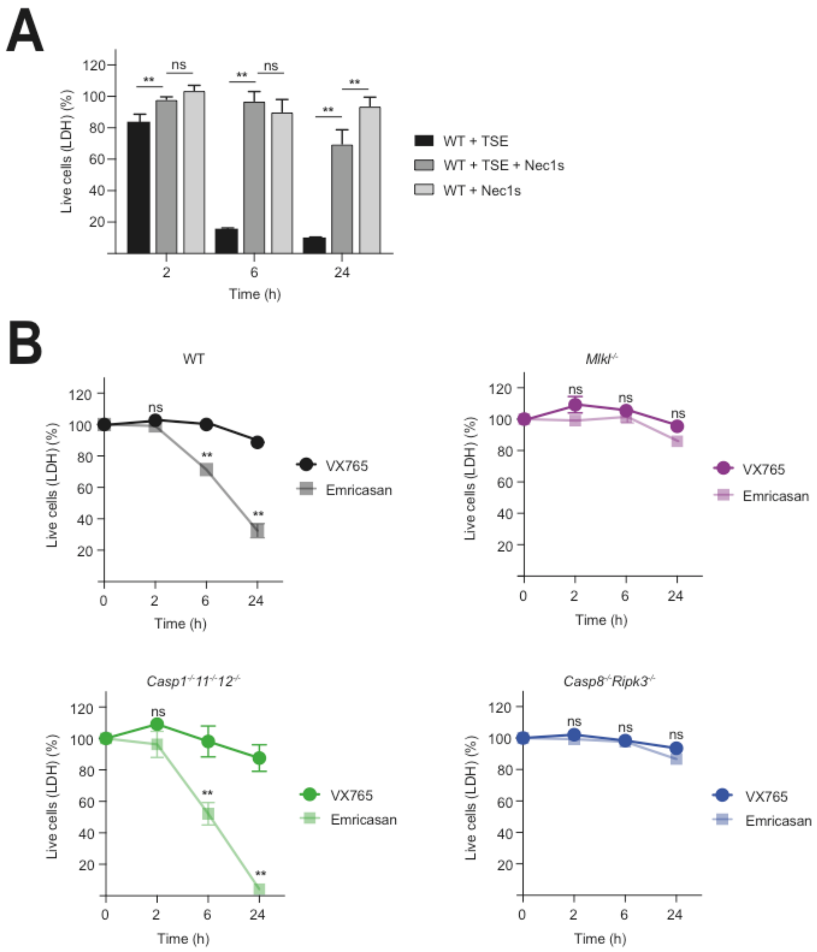

Figure S4 (related to Figures 3 and 4): Impact of RIPK1 Inhibition by Nec1s and Effect of Caspase-1 Inhibition or Inhibition of All Caspases on the Survival of iBMDM.

(A) WT iBMDMs were treated with TNF-α (100 ng/mL) + Birinapant (1 µM) + Emricasan (20 µM) with or without the RIPK1 inhibitor Nec1s (30 µM) and cell death was measured by LDH release. Data pooled from 2 or more experiments. Mean and SEM are shown. **p<0.005; ^{ns}p>0.05=not significant.

(B) LDH release cell death assays of WT, *MLKL*$^{-/-}$, *Casp1*$^{-/-}$;*Casp11*$^{-/-}$;*Casp12*$^{-/-}$, *Casp8*$^{-/-}$;*Ripk3*$^{-/-}$ and *Casp1*$^{-/-}$;*Casp11*$^{-/-}$;*Casp12*$^{-/-}$;*Casp8*$^{-/-}$;*Ripk3*$^{-/-}$ iBMDMs that had been treated with the caspase-1 specific inhibitor, VX-765 (20 µM) or the broad-spectrum caspase inhibitor, Emricasan (20 µM). Data pooled from 2 or more experiments. Mean and SEM are shown. **p<0.005; ^{ns}p>0.05=not significant.

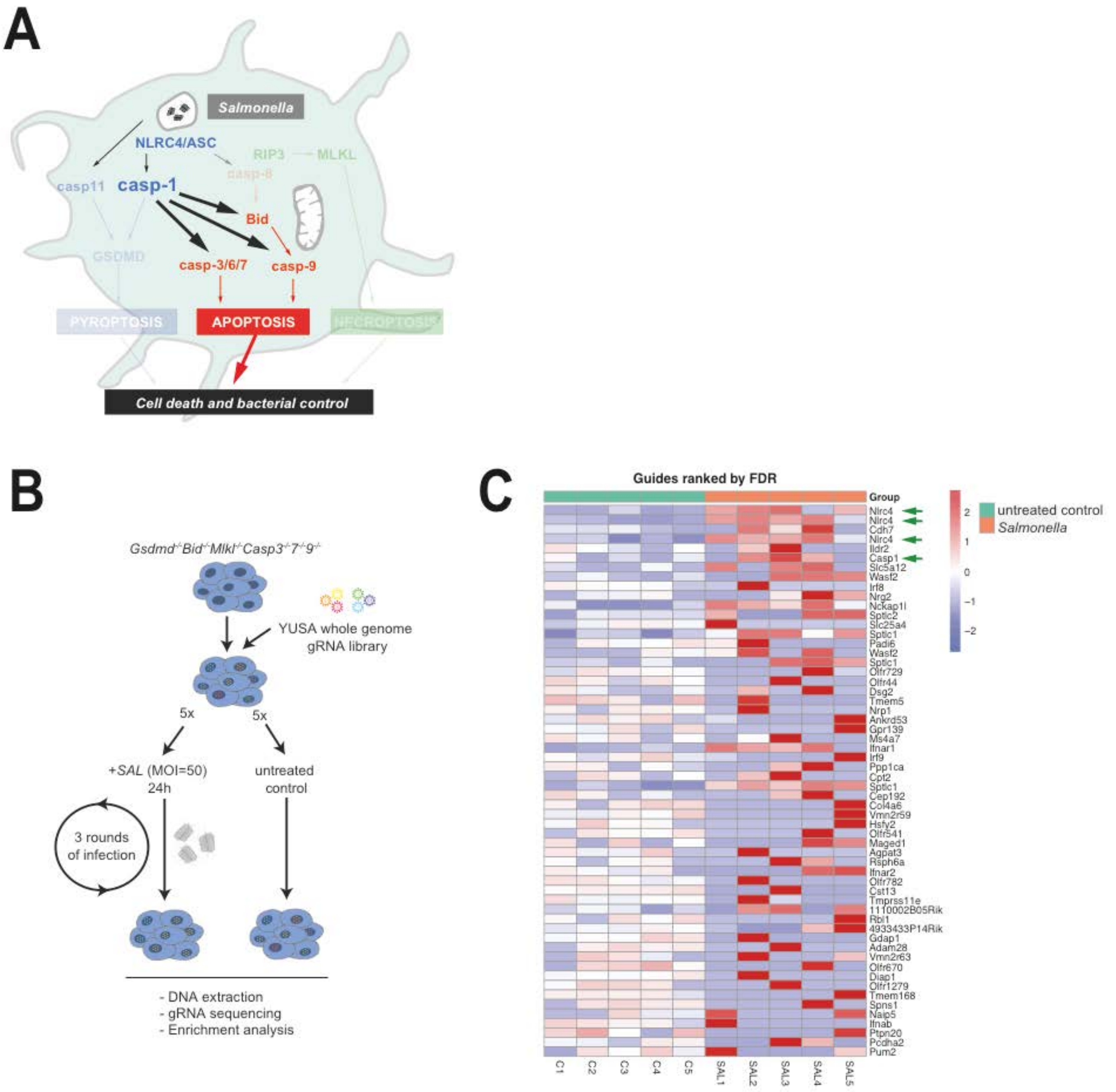

Figure S5 (related to Figures 5 and 6). CRISPR/Cas9 Screen Identifies Caspase-1 and NRLC4 as the Central Drivers of Cell Death in the Absence of all known Downstream Effectors of Cell Killing.

(A) Schematic overview of cell death induction by caspase-1 in the absence of pyroptosis and caspase-8.

(B) Schematic overview of CRISPR/Cas9 whole genome screen: *GsdmD*$^{-/-}$*;Bid*$^{-/-}$*;Mlkl*$^{-/-}$*;Casp3*$^{-/-}$*;Casp7*$^{-/-}$*;Casp9*$^{-/-}$ iBMDMs were transduced with a whole genome sgRNA library (Koike-Yusa et al., 2014) and infected in replicates for three consecutive rounds with *Salmonella* SL1344 (MOI=50). Surviving cells were expanded and subjected to NGS analysis.

(C) A heatmap of the sgRNAs significantly enriched during the CRISPR/Cas9 screen as shown per individual replicate, ranked according to false discovery rate. Green arrowheads indicate sgRNAs for caspase-1 and NLRC4.

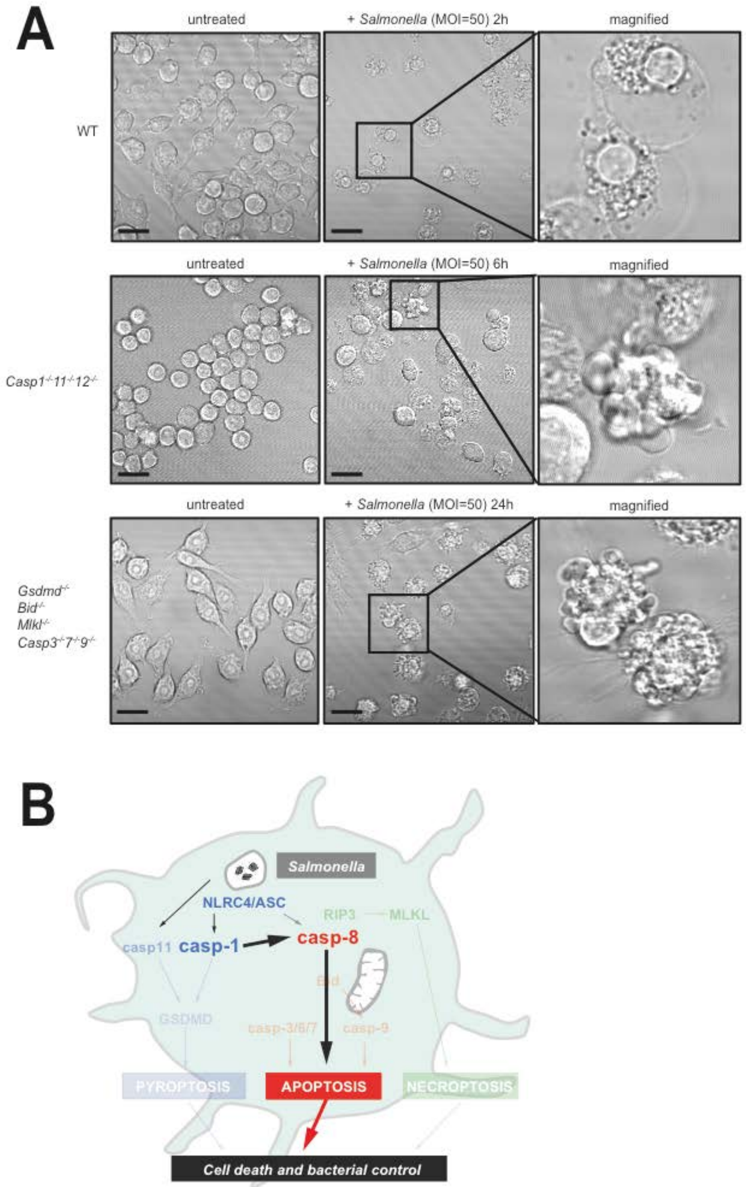

Figure S6 (related to Figures 6 and 7). Macrophages Lacking All Downstream Effectors of Caspase-1 and Caspase-8 Undergo Delayed Apoptotic Cell Death upon *Salmonella* Infection.

(A) WT, *Casp1*$^{-/-}$;*Casp11*$^{-/-}$;*Casp12*$^{-/-}$and *GsdmD*$^{-/-}$;*Bid*$^{-/-}$;*Mlkl*$^{-/-}$;*Casp3*$^{-/-}$; *Casp 7*$^{-/-}$; *Casp 9*$^{-/-}$ iBMDM cells were left untreated or infected with *Salmonella* SL1344 (MOI=50) and analyzed using brightfield microscopy. Scale bar: 20 µm.

(B) Schematic of cell death pathway hierarchy in the absence of all known downstream effectors of apoptosis, pyroptosis and necroptosis
