# Supplementary material for: Flexible Usage and Interconnectivity of Diverse Cell Death Pathways Protect against Intracellular Infection
Source: Immunity. 2020 Sep 15;53(3):533–547.e7. doi: 10.1016/j.immuni.2020.07.004 (PMC7500851; doi:10.1016/j.immuni.2020.07.004)
Supplement: Document S2. Article plus Supplemental Information [file mmc5.pdf]

Article

Immunity

Flexible Usage and Interconnectivity of Diverse Cell Death Pathways Protect against Intracellular Infection

Graphical Abstract

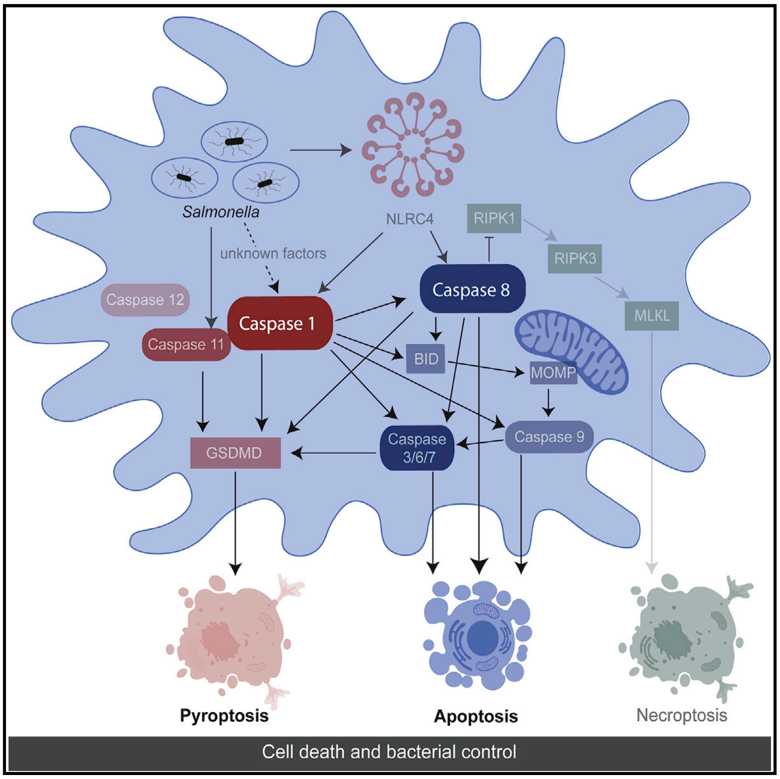

Authors

Marcel Doerflinger, Yexuan Deng, Paul Whitney, ..., Andreas Strasser, Sammy Bedoui, Marco J. Herold

Correspondence

strasser@wehi.edu.au (A.S.), sbedoui@unimelb.edu.au (S.B.), herold@wehi.edu.au (M.J.H.)

In Brief

The clearance of intracellular pathogens requires the killing of infected cells, but it remains unclear why host cells have so many different means of inducing programmed cell death. Doerflinger et al. demonstrate that interconnectivity between pyroptosis and apoptosis involving flexible deployment of caspases ensures control of *Salmonella* infection in mice.

Highlights

- Mice lacking pyroptosis and apoptosis cannot control *Salmonella* infection
- Macrophages lacking pyroptosis and apoptosis resist *Salmonella*-induced killing
- Caspase-1 kills *Salmonella*-infected cells by activating GSDMD, BID, or other caspases
- Caspase-1 and -8 act as cell death executioners when all cell death effectors are lost

Doerflinger et al., 2020, Immunity *53*, 533–547
September 15, 2020 © 2020 The Author(s). Published by Elsevier Inc.
https://doi.org/10.1016/j.immuni.2020.07.004

CellPress

Immunity

CellPress
OPEN ACCESS

Article

Flexible Usage and Interconnectivity of Diverse Cell Death Pathways Protect against Intracellular Infection

Marcel Doerflinger,[1,2,12] Yexuan Deng,[1,3,12] Paul Whitney,[2,4,12] Ranja Salvamoser,[1,2,12] Sven Engel,[2,4] Andrew J. Kueh,[1,2] Lin Tai,[1] Annabell Bachem,[2,4] Elise Gressier,[2,4] Niall D. Geoghegan,[1,2] Stephen Wilcox,[1,2] Kelly L. Rogers,[1,2] Alexandra L. Garnham,[1,2] Michael A. Dengler,[1,2] Stefanie M. Bader,[1] Gregor Ebert,[1,2] Jaclyn S. Pearson,[5,6,7] Dominic De Nardo,[1,2,8] Nancy Wang,[2,4] Chenying Yang,[2,4,11] Milton Pereira,[9,10] Clare E. Bryant,[10] Richard A. Strugnell,[2,4] James E. Vince,[1,2] Marc Pellegrini,[1,2] Andreas Strasser,[1,2,13,14,*] Sammy Bedoui,[2,4,13,*] and Marco J. Herold[1,2,13,*]
[1]The Walter and Eliza Hall Institute of Medical Research, Parkville, VIC, Australia
[2]Department of Medical Biology, University of Melbourne, Parkville, VIC, Australia
[3]The State Key Laboratory of Pharmaceutical Biotechnology, School of Life Sciences, Nanjing University, Nanjing, China
[4]Department of Microbiology and Immunology at the Doherty Institute for Infection and Immunity, The University of Melbourne, Parkville, VIC, Australia
[5]Centre for Innate Immunity and Infectious Diseases, Hudson Institute of Medical Research, Clayton, VIC, Australia
[6]Department of Molecular and Translational Research, Monash University, Clayton, VIC, Australia
[7]Department of Microbiology, Monash University, Clayton, VIC, Australia
[8]Department of Anatomy and Developmental Biology, Monash Biomedicine Discovery Institute, Monash University, Clayton, VIC, Australia
[9]Division of Infectious Diseases and Immunology, University of Massachusetts Medical School, Worcester, MA, USA
[10]University of Cambridge, Cambridge, UK
[11]Present address: Menzies Health Institute Queensland, Griffith University, QLD, Australia
[12]These authors contributed equally
[13]These authors contributed equally
[14]Lead Contact
*Correspondence: strasser@wehi.edu.au (A.S.), sbedoui@unimelb.edu.au (S.B.), herold@wehi.edu.au (M.J.H.)
https://doi.org/10.1016/j.immuni.2020.07.004

SUMMARY

Programmed cell death contributes to host defense against pathogens. To investigate the relative importance of pyroptosis, necroptosis, and apoptosis during *Salmonella* infection, we infected mice and macrophages deficient for diverse combinations of caspases-1, -11, -12, and -8 and receptor interacting serine/threonine kinase 3 (RIPK3). Loss of pyroptosis, caspase-8-driven apoptosis, or necroptosis had minor impact on *Salmonella* control. However, combined deficiency of these cell death pathways caused loss of bacterial control in mice and their macrophages, demonstrating that host defense can employ varying components of several cell death pathways to limit intracellular infections. This flexible use of distinct cell death pathways involved extensive cross-talk between initiators and effectors of pyroptosis and apoptosis, where initiator caspases-1 and -8 also functioned as executioners when all known effectors of cell death were absent. These findings uncover a highly coordinated and flexible cell death system with in-built fail-safe processes that protect the host from intracellular infections.

INTRODUCTION

Metazoans employ different types of programmed cell death (PCD), including apoptosis, necroptosis, and pyroptosis, for the removal of unwanted cells, such as those infected with pathogens (Green, 2019). Apoptosis is executed by so-called effector caspases (caspases-3 and -7, and possibly caspase-6) (Salvesen and Dixit, 1997) that promote cellular fragmentation into apoptotic bodies and engulfment of dying cells by neighboring cells, thus preventing release of intracellular content causing inflammation (Nagata, 2018). Apoptosis can be induced by death receptors such as FAS or TNFR1, which activate caspase-8, or in response to diverse cellular stresses via the intrinsic pathway, which involves BH3-only protein-initiated and Bcl-2-associated protein (BAX) and BCL-2 homologous antagonists killer (BAK)-mediated mitochondrial outer membrane permeabilization (MOMP) (Czabotar et al., 2014). This causes activation of the initiator caspase, caspase-9, and subsequent proteolytic triggering of the effector caspases. Pyroptosis is induced through nucleotide-binding oligomerization domain and leucine-rich repeat-containing receptor (NLR)-dependent activation of caspase-1 or LPS-induced activation of caspase-11 (Lamkanfi and Dixit, 2014; Zhao and Shao, 2016). Ligation of tumor necrosis factor receptor-1 (TNFR1) or Toll-like receptors (TLRs) causes phosphorylation of receptor interacting serine/threonine kinase (RIPK) 1 and RIPK3 to initiate necroptosis

This is an open access article under the CC BY license (http://creativecommons.org/licenses/by/4.0/).

when caspase-8 activity is absent (Ofengeim and Yuan, 2013; Vandenabeele et al., 2010). Pyroptosis and necroptosis are both executed through lysis of the plasma membrane that releases cellular content into the extracellular space, which can elicit pro-inflammatory responses priming the innate as well as the adaptive immune systems.

One important biological function of cellular suicide is to control intracellular pathogens (Jorgensen et al., 2017; Kayagaki et al., 2015; Shi et al., 2015). The killing of infected cells is thought to remove a replicative niche, re-expose the pathogen to extracellular immune effector mechanisms, and make antigens available for triggering pathogen-specific adaptive immune responses. *Salmonella* has been widely used as a model for studying the role of programmed cell death in host defense (Broz et al., 2012; Franchi et al., 2009). This intracellular pathogen can cause typhoid fever, a systemic infection that affects 10−20 million people worldwide and kills >135,000 individuals per annum (Browne et al., 2020). The disease can be modeled by infecting mice with *S.* enterica serovar Typhimurium (Kupz et al., 2014), where spleen and liver are major sites of replication of these bacteria. The primary target of *Salmonella* spp. are phagocytes in which the bacteria survive by repurposing a host-cell-derived membrane compartment into a specialized niche. Phagocytes, such as macrophages, respond to *Salmonella* infection through inflammasome formation involving NLR family apoptosis inhibitory proteins (NAIP)2 or NAIP, and NLRs such as NLRC4 and NLRP3 (Franchi et al., 2009; Miao et al., 2010), which activate caspase-1 (Zhang et al., 2015). Caspase-1 then causes the proteolytic maturation of the inflammatory cytokines interleukin (IL)-1β and IL-18 and release of N-terminal fragments of gasdermin D (GSDMD) proteins that form pores in the cell membrane to elicit pyroptosis. Although these processes appear highly relevant *in vitro*, with caspase-1- or GSDMD-deficient phagocytes resisting *Salmonella*-induced killing (Franchi et al., 2006; Mariathasan et al., 2004), *in vivo* studies suggest that *Salmonella* can be controlled in the absence of inflammasome-driven pyroptosis (Broz et al., 2010). This may reflect the capacity of the host to compensate for the lack of one type of cell death by using another. Such "fail-safe" systems have been hypothesized before (Jorgensen et al., 2017; Rauch et al., 2017; Van Opdenbosch et al., 2017) and may represent the host's response to offset a variety of evasion strategies employed by pathogens to prevent immune recognition (Bedoui et al., 2010). However, very little is known about the organization, regulation, and kinetics of such functional backup in the use of different programmed cell death pathways during host defense against pathogens *in vivo*. Here, we investigated the relative contributions of all initiator and executioner caspases and the cell death effectors they activate to host defense against systemic *Salmonella* infections.

RESULTS

Combined Loss of Caspase-1, Caspase-11, Caspase-12, Caspase-8, and RIPK3 Prevents *Salmonella*-Induced Cell Death and Impairs Bacterial Clearance *In Vivo*

To determine which cell death pathways the host requires for control of intracellular pathogens, we infected C57BL/6 (wild-type: WT) mice with a growth-attenuated strain of *S.* Typhimurium that mirrors the systemic phase of typhoid fever (Kupz et al., 2013, 2014). This infection follows a classical pattern where bacterial growth initially outpaces host defense. By about week 3, bacterial titers reach a peak that is followed by dropping titers and eventual clearance of the bacteria from the host. This type of infection thus allows detailed *in vivo* investigations into the mechanisms that enable *Salmonella* control by innate immune mechanisms over the first 3 weeks of the infection (Kupz et al., 2012, 2013) and T-cell-mediated immune clearance thereafter (Benoun et al., 2018). Consistent with earlier reports using WT strains of *S.* Typhimurium (Broz et al., 2012), we observed slightly elevated bacterial titers in *Casp1*$^{-/-}$*;Casp11*$^{-/-}$ mice 3 weeks post-infection compared to WT controls (Figure 1A), but the lack of pyroptosis did not affect their capacity to clear the bacteria by 12 weeks post-infection. This indicated a minor defect in bacterial control. Exploiting this *in vivo* model of caspase-1 and -11 independent bacterial control, we explored the role of other cell death pathways and their key constituents. We first investigated whether the lack of caspases-1 and -11 was compensated for by caspase-12, given their substantial amino acid similarity and chromosomal co-localization. However, at week 3 post-infection, *Casp1*$^{-/-}$*;Casp11*$^{-/-}$*;Casp12*$^{-/-}$ and *Casp1*$^{-/-}$*;Casp11*$^{-/-}$ mice presented with similar bacterial titers that were slightly higher compared to those observed in WT controls (Figure 1B), revealing that caspase-12 did not play a critical role in bacterial clearance by compensating for the combined absence of caspases-1 and -11.

Caspase-8 has been suggested to coordinate an alternative pathway toward pyroptosis that operates independently of caspases-1 and -11 (Mascarenhas et al., 2017; Orning et al., 2018). This prompted us to investigate the contribution of caspase-8-driven cell death to *Salmonella* control in mice. To prevent the necroptosis-driven embryonic lethality caused by loss of caspase-8, we used *Casp8*$^{-/-}$*;Ripk3*$^{-/-}$ mice (Alvarez-Diaz et al., 2016; Kaiser et al., 2011; Oberst et al., 2011). The combined lack of caspase-8-mediated apoptosis and RIPK3-driven necroptosis did not have significant impact on *Salmonella* titers 3 weeks post-infection (Figure 1B). Mice lacking necroptosis alone (*Mlkl*$^{-/-}$ mice) or those with combined deficiency in pyroptosis and necroptosis (*Casp1*$^{-/-}$*;Casp11*$^{-/-}$*;Casp12*$^{-/-}$*;Ripk3*$^{-/-}$ mice) had no defects in bacterial control until at least 3 weeks post-infection (Figures 1B and S1A). These findings demonstrate that mice with defects in select types of programmed cell death only have minor impairments in their ability to control bacterial replication.

These findings raised the possibility that *in vivo* control of *Salmonella* infection was safeguarded by extensive functional backup between several programmed cell death processes. To investigate this, we generated *Casp1*$^{-/-}$*;Casp11*$^{-/-}$*;Casp12*$^{-/-}$*;Casp8*$^{-/-}$*;Ripk3*$^{-/-}$ mice that are deficient for pyroptosis, death-receptor-induced apoptosis, and necroptosis. These mice had drastically elevated bacterial titers in liver and spleen at both week 2 and 3 post-infection compared to WT animals (Figures 1B and 1C) and had to be sacrificed in accordance with ethical guidelines between 4 and 5 weeks post-infection (Figure 1D). This showed that host defense against *Salmonella* necessitated the activity of at least one of

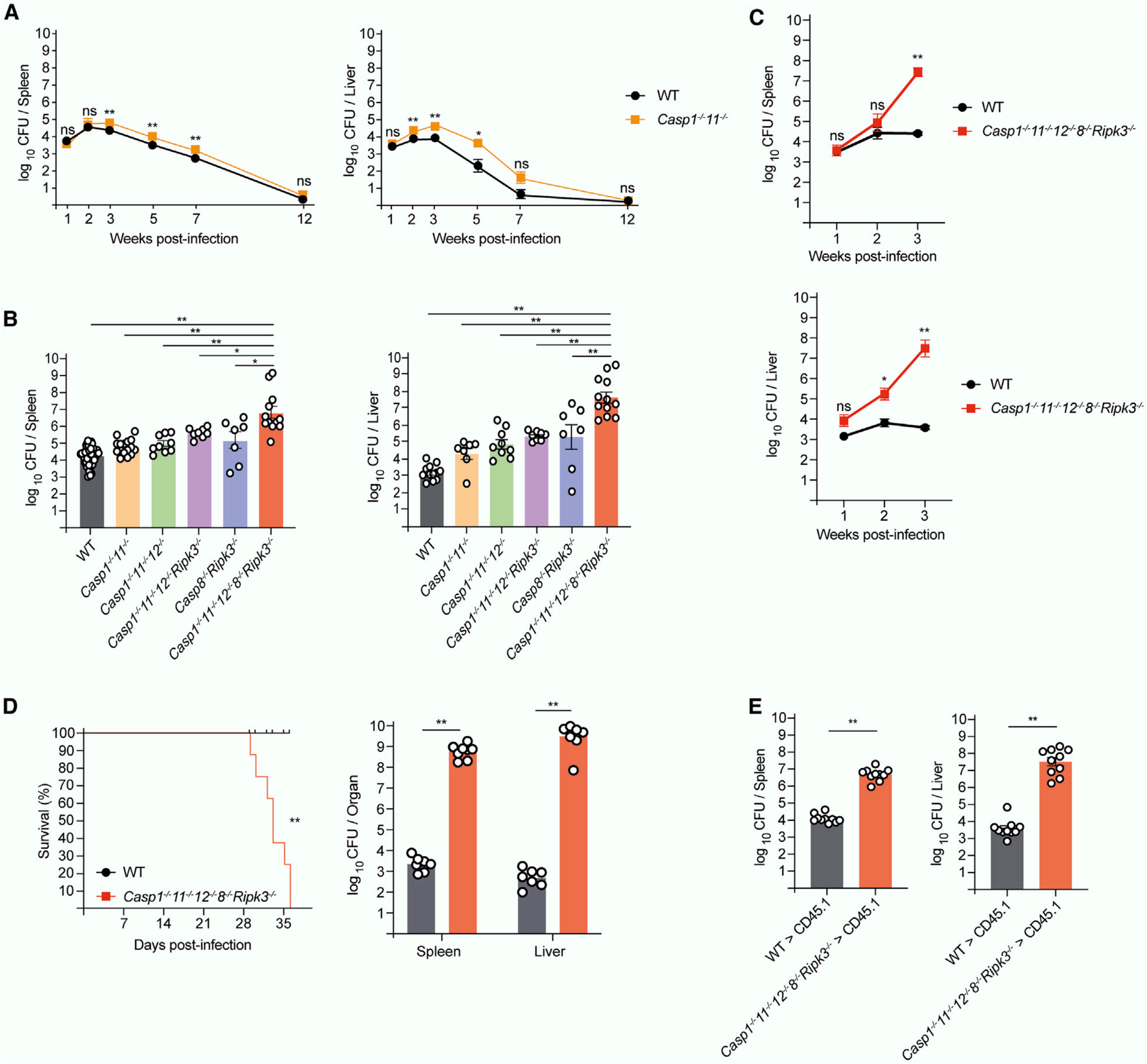

Figure 1. Combined Loss of Caspases-1, -11, -12, and -8 (plus RIPK3) Leads to Lack of Bacterial Control upon *Salmonella* Infection

(A) Bacterial replication over time in WT and *Casp1*$^{-/-}$*;Casp11*$^{-/-}$ mice infected with *Salmonella* Δ*AroA* (200 CFU). n = 10–22 mice per group per time point. Mean and SEM are shown. **p < 0.005, *p < 0.05, ^{ns}p > 0.05 = not significant.

(B) Bacterial loads in spleen and liver of mice of the indicated genotypes 3 weeks post-infection with *Salmonella* Δ*AroA* (200 CFU). n = 7–48 mice per genotype. Mean and SEM are shown. **p < 0.005, *p < 0.05, ^{ns}p > 0.05 = not significant.

(C) Bacterial loads in spleen and liver from mice of the indicated genotypes 1 to 3 weeks post-infection with *Salmonella* Δ*AroA* (200 CFU). n = 3–4 mice per genotype and time point. Mean and SEM are shown. **p < 0.005, *p < 0.05, ^{ns}p > 0.05 = not significant.

(D) Mouse survival curves and corresponding bacterial loads in the spleen and liver at time of sacrifice in WT and *Casp1*$^{-/-}$*;Casp11*$^{-/-}$*;Casp12*$^{-/-}$*;Casp8*$^{-/-}$*;Ripk3*$^{-/-}$ mice infected with *Salmonella* Δ*AroA* (200 CFU). n = 7–8 mice per genotype. Mean and SEM are shown. **p < 0.005.

(E) Bone marrow chimeras of the indicated genotypes were infected with *Salmonella* Δ*AroA* (200 CFU) and culled for analysis of bacterial loads in spleen and liver 3 weeks post-infection. n = 10 mice per group. Mean and SEM are shown. **p < 0.005.

Please also see Figure S1.

these types of programmed cell death pathways and that none of the other known cell death pathways (e.g., intrinsic apoptosis or ferroptosis) were sufficient to ensure control of the infection in their absence. Of note, we observed similar defects in host defense in bone marrow chimeras in which pyroptosis, caspase-8-mediated apoptosis, and necroptosis were only missing from the immune cell compartment (Figure 1E). Therefore, we conclude that *Salmonella* control broke down in *Casp1*$^{-/-}$*;Casp11*$^{-/-}$*;Casp12*$^{-/-}$*;Casp8*$^{-/-}$*;Ripk3*$^{-/-}$ mice because phagocytes could no longer purge the bacteria from their vacuolar compartments by undergoing programmed cell death.

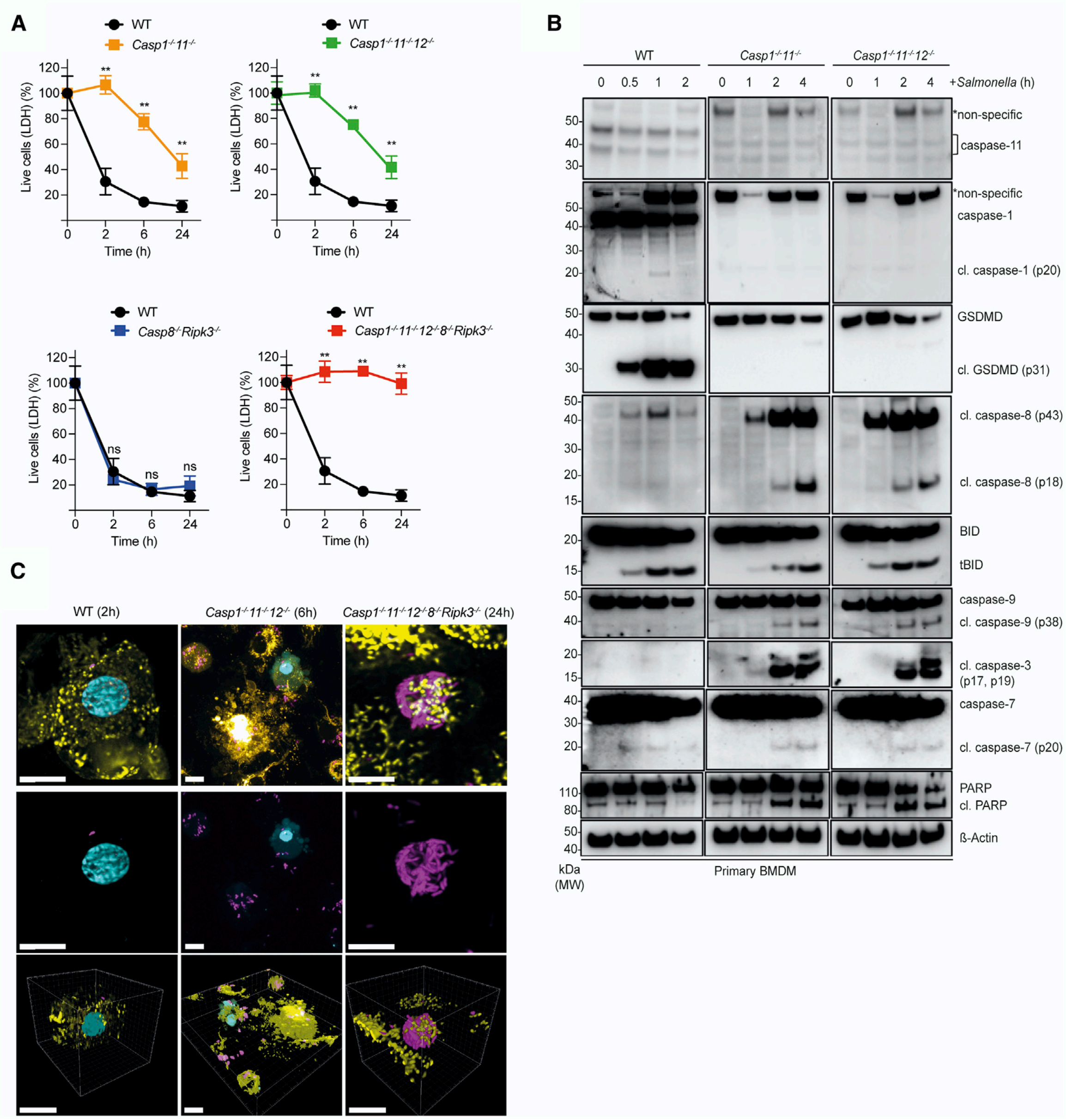

Figure 2. Combined Loss of Caspases-1, -11, -12, and -8 Abrogates the Death of BMDMs upon *Salmonella* Infection

(A) LDH release cell death assay of primary BMDMs of the indicated genotypes after infection with *Salmonella* SL1344 (MOI = 50). Data pooled from two or more experiments. Mean and SEM are shown. $^{**}p < 0.005$, $^{ns}p > 0.05$ = not significant.

(B) BMDMs of the indicated genotypes were infected with *Salmonella* SL1344 (MOI = 50) and cleavage associated with activation of the indicated cell death regulators was analyzed by immunoblotting at the indicated time points. Probing for β-actin served as a loading control.

(C) Confocal or lattice light-sheet imaging of BMDMs of the indicated genotypes after infection with GFP-expressing *Salmonella* (MOI = 50) at the indicated time points. Yellow, membrane; Magenta, *Salmonella*; Cyan, PI. Scale bars: 10 μm.

Please see also Figure S2.

Combined Loss of Caspase-1, Caspase-11, Caspase-12, Caspase-8, and RIPK3 Prevents *Salmonella*-Induced Killing of BMDMs

Previous reports suggest that caspase-8 can induce pyroptosis through proteolytic activation of GSDMD (Mascarenhas et al., 2017). To explore the nature of the cell death induced by caspase-8 upon infection in the absence of the initiators of pyroptosis, we used bone-marrow-derived macrophages (BMDMs) deficient for caspases-1 and -11, or caspases-1, -11, and -12, and infected them with *Salmonella*. As previously reported (Franchi et al., 2006; Mariathasan et al., 2004), *Casp1*$^{-/-}$;*Casp11*$^{-/-}$ BMDMs are protected from *Salmonella*-induced killing at early time points. However, 6 h after infection with *Salmonella*, a substantial fraction of *Casp1*$^{-/-}$;*Casp11*$^{-/-}$ and *Casp1*$^{-/-}$;*Casp11*$^{-/-}$;*Casp12*$^{-/-}$ BMDMs had died (Figure 2A), reiterating that caspase-12 was not critical for the response to

Salmonella infection. The delayed type of *Salmonella*-induced cell death in *Casp1*$^{-/-}$;*Casp11*$^{-/-}$ and *Casp1*$^{-/-}$;*Casp11*$^{-/-}$;*Casp12*$^{-/-}$ BMDMs was unlikely to be due to necroptosis, as we could not detect changes in phosphorylation of MLKL, a hallmark of necroptosis (Figure S1B). Instead, *Casp1*$^{-/-}$;*Casp11*$^{-/-}$ and *Casp1*$^{-/-}$;*Casp11*$^{-/-}$;*Casp12*$^{-/-}$ BMDMs displayed hallmarks of apoptosis, including cleavage of Poly (ADP-ribose) polymerase (PARP) as well as caspases-3, -7, -8, and -9 and BH3 interacting-domain agonist (BID) (Figure 2B). This extends a previous report showing that anthrax lethal toxin can induce a NLRP1-dependent form of cell death with features of apoptosis in cells lacking caspase-1 (Van Opdenbosch et al., 2017). Lattice light-sheet microscopy revealed nuclear condensation and plasma membrane blebbing, which was consistent with apoptotic death of *Salmonella*-infected *Casp1*$^{-/-}$;*11*$^{-/-}$;*12*$^{-/-}$ BMDMs and contrasted with the pyroptotic death observed in *Salmonella*-infected WT BMDMs (Figure 2C; Videos S1, S2, and S3). Notably, combined loss of caspase-8 plus RIPK3 did not impair *Salmonella*-induced cell killing, as *in vitro*-infected *Casp8*$^{-/-}$;*Ripk3*$^{-/-}$ BMDMs died with kinetics that were indistinguishable from WT cells, with both undergoing pyroptosis (Figure 2A). This was consistent with the observation that the combined loss of caspase-8 and RIPK3 did not impair bacterial control *in viv*o until at least 3 weeks post-infection (Figure 1B). These findings indicate that although caspase-8 was dispensable for the early pyroptotic cell death upon *Salmonella* infection, caspase-8-driven apoptosis, rather than caspase-8-mediated pyroptosis or RIPK3- and MLKL-driven necroptosis, was responsible for the delayed type of cell death observed in *Casp1*$^{-/-}$;*Casp11*$^{-/-}$;*Casp12*$^{-/-}$ BMDMs. *Casp1*$^{-/-}$;*Casp11*$^{-/-}$;*Casp12*$^{-/-}$;*Casp8*$^{-/-}$;*Ripk3*$^{-/-}$ BMDMs were not only profoundly resistant to *Salmonella*-induced killing *in vitro* (Figure 2A), but also contained large numbers of bacteria (Figure 2C). This resistance to *Salmonella*-induced killing was not due to a general defect in cell death, as *Casp1*$^{-/-}$;*Casp11*$^{-/-}$;*Casp12*$^{-/-}$;*Casp8*$^{-/-}$;*Ripk3*$^{-/-}$ BMDMs could still be killed through the intrinsic pathway of apoptosis by treatment with BH3 mimetic drugs, as shown by lactate dehydrogenase (LDH) release and activation of the apoptosis effector BAX (Figures S2A and S2B). Collectively, these findings uncover a backup system that enables the host to flexibly deploy different types of programmed cell death for the control of the intracellular pathogen *Salmonella*.

Immortalized BMDMs Facilitate Unraveling of the Diverse Cell Death Mechanisms Induced upon *Salmonella* Infection

To gain a deeper understanding of this complex system of functional backup between different cell death processes, we employed our CRISPR-Cas9 gene editing platform (Aubrey et al., 2015) to identify the initiators and effectors critical for the respective types of cell death upon *Salmonella* infection. We used immortalized BMDMs (iBMDMs) for these experiments, which exhibited comparable responses to *Salmonella* infection as primary BMDMs (Figures 3A and 3B). While the combined loss of caspases-1, -11, and -12 delayed *Salmonella*-induced killing of iBMDMs, the loss of both caspase-8 and RIPK3 had no impact, and cells died in a manner comparable to WT cells (Figure 3A). Only the combined absence of caspases-1, -11, -12, and -8 and RIPK3 completely blocked *Salmonella*-infection-induced killing of iBMDMs (Figure 3A). The *Casp1*$^{-/-}$;*Casp11*$^{-/-}$;*Casp12*$^{-/-}$;*Casp8*$^{-/-}$;*Ripk3*$^{-/-}$ iBMDMs still underwent cell death in response to treatment with combinations of BH3 mimetics or etoposide, and this was accompanied by activation of the apoptosis effector BAX (Figures S3A and S3B), as was the case for primary BMDMs (Figures S2A and S2B). These results validate iBMDMs as useful tools to unravel the molecular requirements of the diverse cell death pathways induced upon *Salmonella* infection. We also noted in these experiments that *Casp1*$^{-/-}$;*Casp11*$^{-/-}$;*Casp12*$^{-/-}$ iBMDMs showed more prominent processing of caspase-8 following *Salmonella* infection compared to WT iBMDMs and that this was accompanied by classical markers of apoptosis, such as cleavage of BID and caspases-3, -7, and -9 (Figures 2B and 3B). *Casp1*$^{-/-}$;*Casp11*$^{-/-}$;*Casp12*$^{-/-}$ cells infected with *Salmonella* in the absence or presence of the highly selective RIPK1 inhibitor, Nec1s (which protected against necroptosis induced by TNF-α+Birinapant+Emricasan; Figure S4A), still underwent cell death and showed cleavage of caspases-3 and -8 and BID (Figures 3C and 3D). These findings indicate that the activation of caspase-8 upon infection with *Salmonella* was unlikely to depend on RIPK1-dependent ripoptosomes (Tenev et al., 2011).

Caspase-11 Can Partially Compensate for the Combined Loss of Caspase-1 and Caspase-8

To identify which of the initiator caspases were required for *Salmonella*-induced cell death, we treated WT, *Casp1*$^{-/-}$;*Casp11*$^{-/-}$;*Casp12*$^{-/-}$, and *Casp8*$^{-/-}$;*Ripk3*$^{-/-}$ iBMDMs with different caspase inhibitors and examined their cell death responses (Figures 4A and S4B). The broad-spectrum caspase inhibitor Emricasan stalled the early cell death response in *Salmonella*-infected WT iBMDMs, similar to what was seen in *Casp1*$^{-/-}$;*Casp11*$^{-/-}$;*Casp12*$^{-/-}$ BMDMs, but later on, these cells also died (Figure 4A). We hypothesized that the late death was due to necroptosis caused by the blockade of caspase-8, and we confirmed this by showing that treatment with Emricasan killed WT but not MLKL-deficient iBMDMs (Figure S4B). Accordingly, Emricasan completely blocked *Salmonella*-induced killing in *MLKL*$^{-/-}$ iBMDMs (Figure 4A). This demonstrated that caspase activity was required for *Salmonella*-induced cell killing. Inhibition of caspase-1 by VX765 delayed but did not abrogate the killing of *Salmonella*-infected WT, *MLKL*$^{-/-}$, and *Casp8*$^{-/-}$;*Ripk3*$^{-/-}$ cells (Figures 4A and S4A). These pharmacological approaches validated the above-described observations from the genetic studies and reaffirmed that the functional differences are not the consequence of longer-term adaptations of cells driven by the loss of the genes of interest.

Inhibition of caspase-1 activity by VX765 or genetic deletion of caspase-1 in *Casp8*$^{-/-}$;*Ripk3*$^{-/-}$ iBMDMs only reduced *Salmonella*-induced killing but did not afford the profound protection seen in *Casp1*$^{-/-}$;*Casp11*$^{-/-}$;*Casp12*$^{-/-}$;*Casp8*$^{-/-}$;*Ripk3*$^{-/-}$ iBMDMs (Figures 4A and 4B). We therefore hypothesized that caspase-11 may provide a backup mechanism for cell killing when caspases-1 and -8 are both absent or inhibited (Man et al., 2017; Ng and Monack, 2013). To investigate this, we generated *Casp1*$^{-/-}$;*Casp8*$^{-/-}$;*Ripk3*$^{-/-}$ iBMDMs and compared their death kinetics upon *Salmonella* infection to those of *Casp1*$^{-/-}$;*Casp11*$^{-/-}$;*Casp12*$^{-/-}$;*Casp8*$^{-/-}$;*Ripk3*$^{-/-}$ cells. *Casp1*$^{-/-}$;*Casp8*$^{-/-}$;*Ripk3*$^{-/-}$ iBMDMs responded to the infection

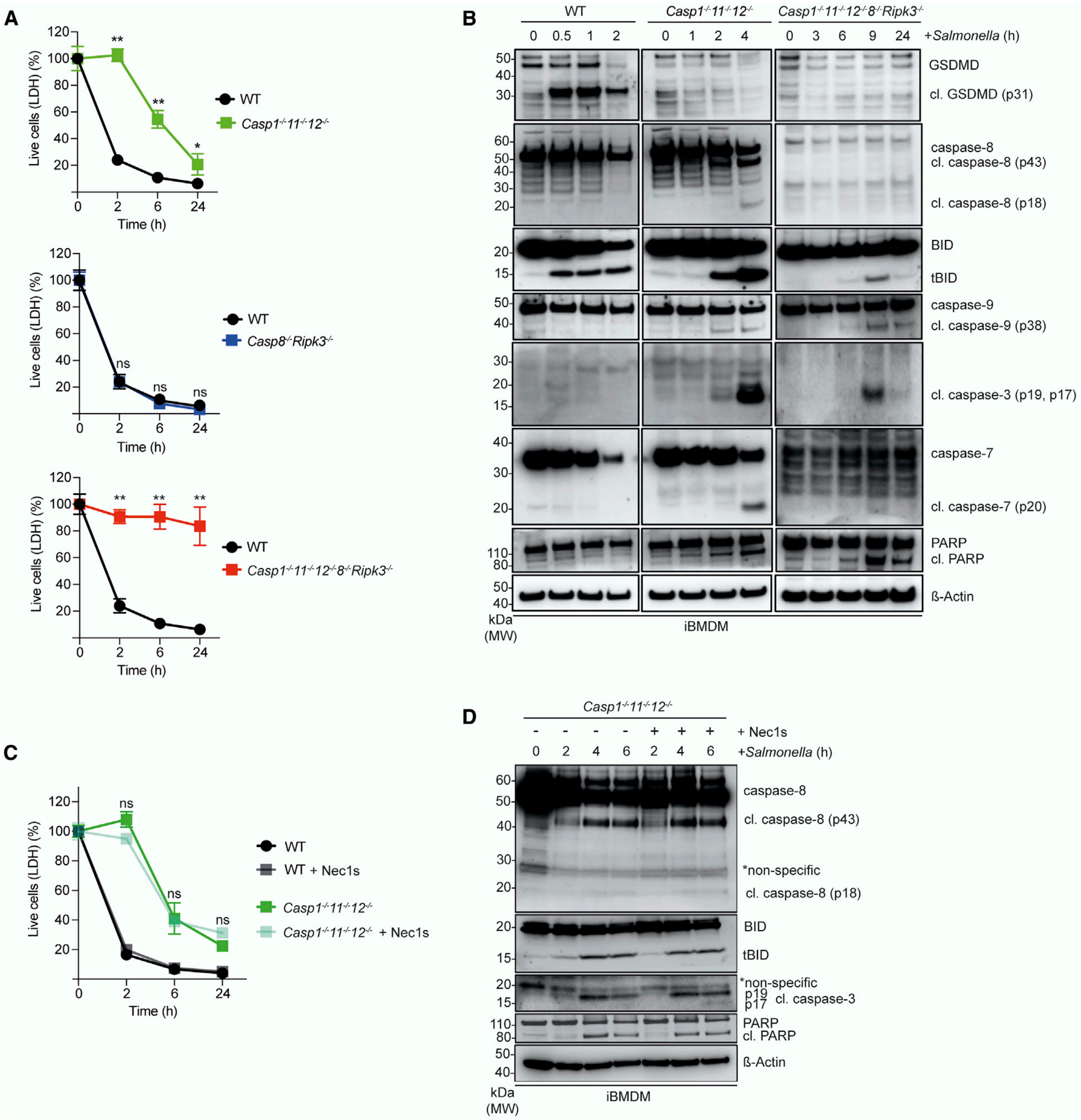

Figure 3. Caspase-8-Mediated Apoptosis Is the Default Backup Mechanism when Caspases-1- and 11-Mediated Pyroptosis Is Disabled in *Salmonella*-Infected iBMDMs

(A) LDH release cell death assay of iBMDMs of the indicated genotypes after infection with *Salmonella* SL1344 (MOI = 50). Data pooled from two or more experiments. Mean and SEM are shown. $^{**}p < 0.005$, $^{ns}p > 0.05$ = not significant.

(B) Immunoblot analysis of the indicated proteins in iBMDMs of the indicated genotypes after infection with *Salmonella* SL1344 (MOI = 50). Probing for β-actin served as a loading control.

(C) LDH release cell death assay of *Salmonella* SL1344 (MOI = 50) -infected WT and *Casp1*$^{-/-}$;*Casp11*$^{-/-}$;*Casp12*$^{-/-}$ iBMDMs that had been left untreated or treated with the RIPK1 inhibitor, Nec1s (30 μM). Data pooled from two or more experiments. Mean and SEM are shown. $^{**}p < 0.005$, $^{ns}p > 0.05$ = not significant.

(D) Immunoblot analysis of the indicated proteins in *Salmonella* SL1344 (MOI = 50) -infected WT and *Casp1*$^{-/-}$;*Casp11*$^{-/-}$;*Casp12*$^{-/-}$ iBMDMs that had been left untreated or treated with the RIPK1 inhibitor, Nec1s (30 μM). Probing for β-actin served as a loading control.

Please see also Figures S3 and S4.

CellPress
OPEN ACCESS

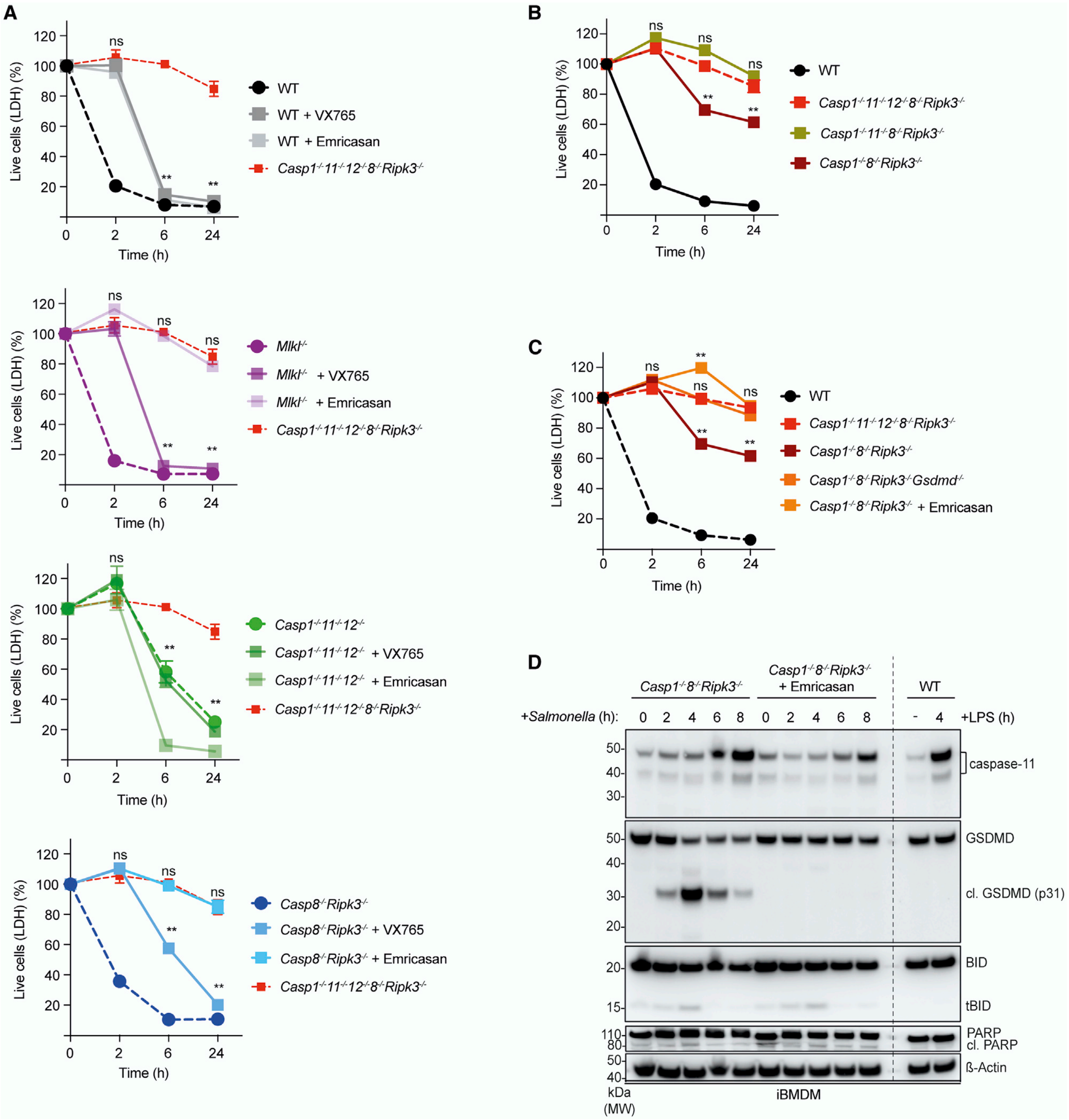

Figure 4. Caspase-11 Can Compensate for the Loss of Caspases-1 and -8 to Ensure GSDMD-Mediated Killing of *Salmonella*-Infected Cells

(A) LDH release cell death assay of *Salmonella* SL1344 (MOI = 50) -infected iBMDMs of the indicated genotypes that had been left untreated or treated with VX-765 or Emricasan. Data pooled from two or more experiments. Mean and SEM are shown. $^{**}p < 0.005$, $^{ns}p > 0.05$ = not significant.

(B) LDH release cell death assays of iBMDMs of the indicated genotypes that had been infected with *Salmonella* SL1344 (MOI = 50). Data pooled from two or more experiments. Mean and SEM are shown. $^{**}p < 0.005$, $^{ns}p > 0.05$ = not significant.

(C) LDH release cell death assays of *Salmonella*-infected iBMDMs of the indicated genotypes or *Casp1*$^{-/-}$;*Casp8*$^{-/-}$;*Ripk3*$^{-/-}$ iBMDMs that had been left untreated or treated with Emricasan and infected with *Salmonella* SL1344 (MOI = 50). Data pooled from two or more experiments. Mean and SEM are shown. $^{**}p < 0.005$, $^{ns}p > 0.05$ = not significant.

(D) Immunoblot analysis of caspase-11, GSDMD, BID, and PARP in *Casp1*$^{-/-}$;*Casp8*$^{-/-}$;*Ripk3*$^{-/-}$ iBMDMs that had been left untreated or treated with Emricasan and infected with *Salmonella* SL1344 (MOI = 50). WT iBMDMs that had been left untreated or treated with LPS for 4 h were used as a control for the induction of caspase-11. Probing for β-actin served as a loading control.

Please see also Figure S4.

by upregulation of caspase-11 and underwent cell death, although this killing was less effective with ∼60%–70% of the cells surviving the bacterial assault (Figures 4B and 4D). *Casp1*$^{-/-}$;*Casp11*$^{-/-}$;*Casp8*$^{-/-}$;*Ripk3*$^{-/-}$ iBMDMs were as resistant to *Salmonella*-induced killing as the *Casp1*$^{-/-}$;*Casp11*$^{-/-}$;*Casp12*$^{-/-}$;*Casp8*$^{-/-}$;*Ripk3*$^{-/-}$ cells (Figure 4B). This revealed again that caspase-12 did not contribute notably to cell death caused by infection with this intracellular pathogen. The broad-spectrum caspase inhibitor Emricasan completely blocked the killing of *Salmonella*-infected *Casp1*$^{-/-}$;*Casp8*$^{-/-}$;*Ripk3*$^{-/-}$ iBMDMs and appearance of the activated form of GSDMD, the critical effector of pyroptosis (Figures 4C and 4D). Notably, *Casp1*$^{-/-}$;*Casp8*$^{-/-}$;*Ripk3*$^{-/-}$ iBMDMs infected with *Salmonella* did not exhibit markers of apoptosis such as cleaved BID or PARP (Figure 4D), and the additional deletion of GSDMD rendered *Casp1*$^{-/-}$;*Casp8*$^{-/-}$;*Ripk3*$^{-/-}$ iBMDMs fully resistant to *Salmonella*-induced killing (Figure 4C). Thus, although caspase-11 can contribute to the killing of *Salmonella*-infected cells, with its effects uncovered by the absence of caspases-1 and -8, the protracted kinetics and exclusive dependence on GSDMD suggest a comparatively minor role for caspase-11 in the backup system governing *Salmonella*-induced killing.

Caspase-1 Orchestrates a Wide Range of Diverse Cell-Death-Inducing Processes with Plasticity

Although our data revealed an important role for caspase-8 in compensating for the lack of pyroptosis, it was also evident that host defense was intact in mice lacking both caspase-8 and RIPK3 (Figure 1B). This suggests that there may not only be redundancy among the different cell death pathways, but that individual components of these processes could possibly be employed in more than one pathway. With this in mind, we reasoned that the lack of caspase-8-mediated apoptosis might be compensated for by caspase-1. To investigate this, we deleted *Gsdmd* to prevent caspases-1 and -11 from triggering pyroptosis and also eliminated *Casp8* and *Ripk3* from iBMDMs. Cells lacking these essential components of apoptosis and necroptosis, as well as being unable to execute pyroptosis, still died upon *Salmonella* infection with kinetics that were indistinguishable from *Gsdmd*$^{-/-}$ iBMDMs (Figure 5A). We noted that *Salmonella*-infected *Gsdmd*$^{-/-}$;*Casp8*$^{-/-}$;*Ripk3*$^{-/-}$ iBMDMs not only had active caspase-1 as predicted, but also contained cleaved BID (tBID) and caspases-3, -7, and -9 (Figure 5B). This indicated that caspase-1 may trigger caspase-8-independent apoptosis via BID-driven, BAX- or BAK-mediated MOMP, and the resulting activation of caspase-9, thereby stimulating the effector caspases-3 and -7. This is consistent with recent reports (Heilig et al., 2020; Tsuchiya et al., 2019) and suggests that caspase-1 can induce apoptosis by bypassing caspase-8 through the mitochondrial amplification loop. However, preventing MOMP through the combined deletion of *Bax* and *Bak* in *Gsdmd*$^{-/-}$;*Casp8*$^{-/-}$;*Ripk3*$^{-/-}$ iBMDMs did not have the predicted effect of completely blocking *Salmonella*-induced cell killing. Instead, *Gsdmd*$^{-/-}$;*Casp8*$^{-/-}$;*Ripk3*$^{-/-}$;*Bax*$^{-/-}$;*Bak*$^{-/-}$ iBMDMs still died upon *Salmonella* infection, containing cleaved caspases-3 and -7 (Figures 5A and 5B), indicating that the combined absence of BAX and BAK could be compensated for by rewiring the cell in a manner that allowed for caspase-1 to trigger the executioner caspases-3 and -7 independently of MOMP. Ablating this alternative cell death circuit by deleting caspases-3 and -7 was yet again not sufficient to prevent cell death, as *Gsdmd*$^{-/-}$;*Casp8*$^{-/-}$;*Ripk3*$^{-/-}$;*Casp3*$^{-/-}$;*Casp7*$^{-/-}$ iBMDMs still died upon *Salmonella* infection, although this occurred with slower kinetics and lower efficiency compared to *Gsdmd*$^{-/-}$;*Casp8*$^{-/-}$;*Ripk3*$^{-/-}$;*Bax*$^{-/-}$;*Bak*$^{-/-}$ iBMDMs (Figure 5C). The fact that *Gsdmd*$^{-/-}$;*Casp8*$^{-/-}$;*Ripk3*$^{-/-}$;*Casp3*$^{-/-}$;*Casp7*$^{-/-}$ iBMDMs still contained cleaved BID and caspases-1 and -9 suggested that tBID and caspase-9 activated by caspase-1 could overcome the lack of the executioner caspases to ensure killing of infected cells (Figure 5D). Additional deletion of BID from *Gsdmd*$^{-/-}$;*Casp8*$^{-/-}$;*Ripk3*$^{-/-}$;*Casp3*$^{-/-}$;*Casp6*$^{-/-}$;*Casp7*$^{-/-}$ iBMDMs did not render the cells fully resistant to *Salmonella*-induced death. This raised the prospect that caspase-1 could directly activate caspase-9, which then acted as an effector caspase rather than an initiator caspase (Figure 5E). Of note, the deletion of caspase-9 from *Gsdmd*$^{-/-}$;*Casp8*$^{-/-}$;*Ripk3*$^{-/-}$;*Bid*$^{-/-}$;*Mlkl*$^{-/-}$;*Casp3*$^{-/-}$;*Casp7*$^{-/-}$ iBMDMs finally reproduced the profound resistance to *Salmonella*-induced killing observed in *Casp1*$^{-/-}$;*Casp11*$^{-/-}$;*Casp12*$^{-/-}$;*Casp8*$^{-/-}$;*Ripk3*$^{-/-}$ iBMDMs (Figure 5F). These findings reveal a substantial degree of plasticity with which caspase-1 can orchestrate the use of diverse cell-death-inducing processes to kill *Salmonella*-infected iBMDMs. Caspase-1 can bypass caspase-8 by initiating apoptosis through mitochondrial amplification and can even circumvent the need for MOMP by activating caspases-3, -7, and -9 directly (see schematic shown in Figure S5A). This shows that many core components widely believed to be essential for apoptosis can be bypassed and that the resulting re-routing of the cell death machinery provides various alternative processes for the killing of pathogen-infected cells.

Caspase-1 Has a Central Role as Both a Cell Death Inducer and Executioner

Our findings provided novel insights into the role of caspase-1 and its capacity to compensate for the lack of caspase-8. However, it is important to note that *Casp1*$^{-/-}$;*Casp11*$^{-/-}$ and *Casp1*$^{-/-}$;*Casp11*$^{-/-}$;*Casp12*$^{-/-}$ mice nonetheless effectively controlled *Salmonella* infection (Figure 1B). This indicates yet another form of compensation whereby caspase-1 can also be functionally replaced, which likely involved caspase-8, as suggested by the observations that *Casp1*$^{-/-}$;*Casp11*$^{-/-}$;*Casp12*$^{-/-}$;*Casp8*$^{-/-}$;*Ripk3*$^{-/-}$ mice were unable to clear bacteria and that BMDMs derived from these animals failed to undergo cell death upon infection (Figures 1B and 1C). We therefore tested the hypothesis that deleting the death effectors activated by both caspases-1 and -8, i.e., GSDMD, BID, MLKL, and caspases-3, -6, -7, and possibly also -9, would recapitulate the profound resistance to *Salmonella*-induced killing observed in *Casp1*$^{-/-}$;*Casp11*$^{-/-}$;*Casp12*$^{-/-}$;*Casp8*$^{-/-}$;*Ripk3*$^{-/-}$ iBMDMs. However, upon infection with *Salmonella*, both *Gsdmd*$^{-/-}$;*Bid*$^{-/-}$;*Mlkl*$^{-/-}$;*Casp3*$^{-/-}$;*Casp7*$^{-/-}$;*Casp9*$^{-/-}$ and *Gsdmd*$^{-/-}$;*Bid*$^{-/-}$;*Mlkl*$^{-/-}$;*Casp3*$^{-/-}$;*Casp6*$^{-/-}$;*Casp7*$^{-/-}$;*Casp9*$^{-/-}$ iBMDMs underwent substantial cell death, although this was delayed compared to WT iBMDMs (Figure 6A). To identify the most potent driver(s) of this unexpected cell killing caused by *Salmonella*, we performed a genome-wide CRISPR-Cas9 screen (Figure S5A). We transduced Cas9 expressing *Gsdmd*$^{-/-}$;*Bid*$^{-/-}$;*Mlkl*$^{-/-}$;*Casp3*$^{-/-}$;*Casp7*$^{-/-}$;*Casp9*$^{-/-}$ iBMDMs with a whole-genome single guide

CellPress
OPEN ACCESS

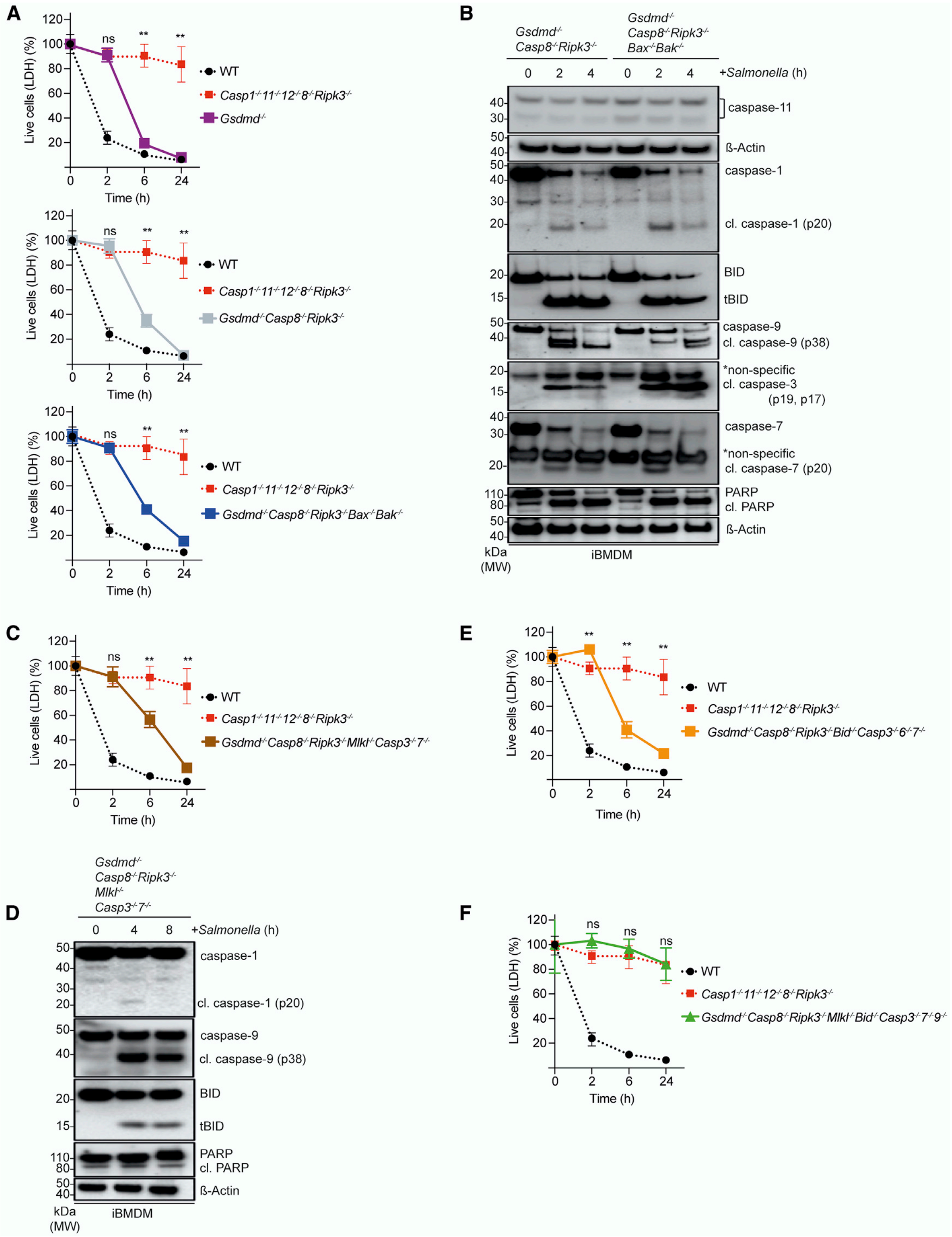

Figure 5. Caspase-1 Can Activate Caspases-3, -7, and -9 Independently of Caspase-8 and BID

(A) LDH release cell death assays of iBMDMs of the indicated genotypes that had been infected with *Salmonella* SL1344 (MOI = 50). Data pooled from two or more experiments. Mean and SEM are shown. $^{**}p < 0.005$, $^{ns}p > 0.05$ = not significant.

(B) iBMDMs of the indicated genotypes were infected with *Salmonella* SL1344 (MOI = 50) and cleavage associated with activation of the indicated cell death proteins was analyzed by immunoblotting at the indicated time points. Probing for β-actin served as a loading control.

(C) LDH release cell death assays of iBMDMs of the indicated genotypes that had been infected with *Salmonella* SL1344 (MOI = 50). Data pooled from two or more experiments. Mean and SEM are shown. $^{**}p < 0.005$, $^{ns}p > 0.05$ = not significant.

(D) *GsdmD*$^{-/-}$;*Casp8*$^{-/-}$;*Ripk3*$^{-/-}$;*Mlkl*$^{-/-}$;*Casp3*$^{-/-}$;*Casp7*$^{-/-}$ iBMDMs were infected with *Salmonella* SL1344 (MOI = 50) and expression and cleavage associated with activation of the indicated cell death proteins was analyzed by immunoblotting at the indicated time points. Probing for β-actin served as a loading control.

(legend continued on next page)

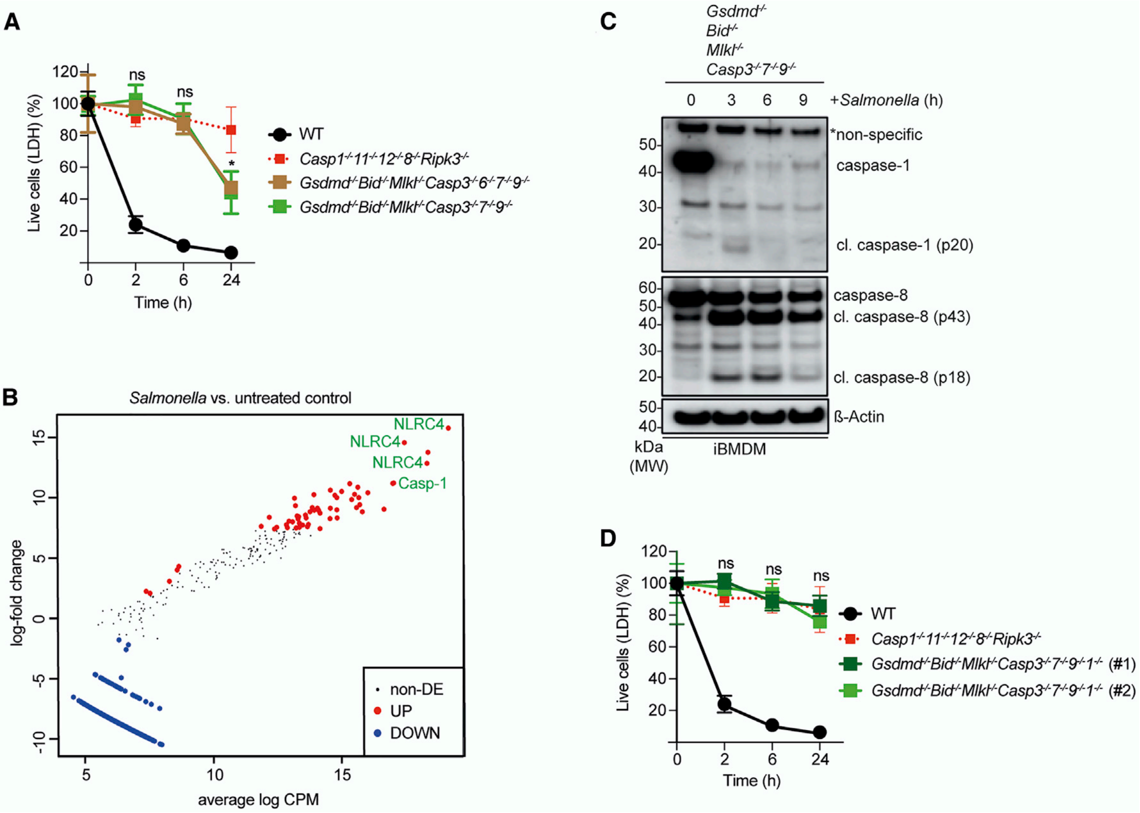

Figure 6. CRISPR Screen Reveals a Central Role for Caspase-1 in Mediating *Salmonella*-Infection-Induced Cell Death Independent of All Known Downstream Effectors of Cell Killing

(A) LDH release cell death assays of iBMDMs of the indicated genotypes that had been infected with *Salmonella* SL1344 (MOI = 50). Data pooled from two or more experiments. Mean and SEM are shown. *p < 0.05, ^{ns}p > 0.05 = not significant.

(B) *GsdmD*$^{-/-}$;*Bid*$^{-/-}$;*Mlkl*$^{-/-}$;*Casp3*$^{-/-}$;*Casp7*$^{-/-}$;*Casp9*$^{-/-}$ iBMDM whole genome CRISPR-Cas9 screen mean-difference (MD) plot showing log-fold change versus average log counts per million (CPM) after three rounds of infection with *Salmonella* SL1344 (MOI = 50) (please also see Figures S5A and S5B).

(C) *GsdmD*$^{-/-}$;*Bid*$^{-/-}$;*Mlkl*$^{-/-}$;*Casp3*$^{-/-}$;*Casp7*$^{-/-}$;*Casp9*$^{-/-}$ iBMDMs were infected with *Salmonella* SL1344 (MOI = 50) and cleavage associated with activation of caspases-1 and -8 was analyzed by immunoblotting at the indicated time points. Probing for β-actin served as a loading control.

(D) LDH release cell death assays of WT, *Casp1*$^{-/-}$;*Casp11*$^{-/-}$;*Casp12*$^{-/-}$;*Casp8*$^{-/-}$;*Ripk3*$^{-/-}$, and two independent clones (#1 and #2) of *GsdmD*$^{-/-}$;*Bid*$^{-/-}$;*Mlkl*$^{-/-}$;*Casp3*$^{-/-}$;*Casp7*$^{-/-}$;*Casp9*$^{-/-}$;*Casp1*$^{-/-}$ iBMDMs that had been infected with *Salmonella* SL1344 (MOI = 50). Data pooled from two or more experiments. Mean and SEM are shown. ^{ns}p > 0.05 = not significant.

Please see also Figure S6.

RNA (sgRNA) library (Koike-Yusa et al., 2014) and stringently enriched for sgRNAs that promoted cell survival after *Salmonella* infection by repeating the infection and selection procedure three times. Amplicon sequencing of the sgRNAs enriched in the surviving cells identified caspase-1 and its activator NLRC4 (Figures 6B and S5B), suggesting the possibility that the initiator caspase-1 could kill *Salmonella*-infected iBMDMs directly (i.e., acting not only as an initiator but also as an effector caspase). This would also explain why cells still died in the absence of pore formation (GSDMD) and all other effectors that are known to function downstream of caspase-1. Consistent with such a role for caspase-1, immunoblot analysis revealed processing of both caspase-1 and caspase-8 in *Salmonella*-infected *Gsdmd*$^{-/-}$;*Bid*$^{-/-}$;*Mlkl*$^{-/-}$;*Casp3*$^{-/-}$;*Casp7*$^{-/-}$;*Casp9*$^{-/-}$ iBMDMs (Figure 6C). Deletion of caspase-1 in *Gsdmd*$^{-/-}$;*Bid*$^{-/-}$;*Mlkl*$^{-/-}$;*Casp3*$^{-/-}$;*Casp7*$^{-/-}$;*Casp9*$^{-/-}$ iBMDMs rendered these cells fully resistant to *Salmonella*-induced killing (comparable to *Casp1*$^{-/-}$;*Casp11*$^{-/-}$;*Casp12*$^{-/-}$;*Casp8*$^{-/-}$;*Ripk3*$^{-/-}$ cells) despite the presence of caspase-8 (Figure 6D). Together with our demonstration that *Gsdmd*$^{-/-}$;*Ripk3*$^{-/-}$;*Bid*$^{-/-}$;*Mlkl*$^{-/-}$;*Casp3*$^{-/-}$;*Casp7*$^{-/-}$;*Casp9*$^{-/-}$ iBMDMs in which caspase-8 was additionally deleted (Figure 5F) were also fully resistant to *Salmonella*-induced killing, these findings indicate that cell death under these circumstances was only possible when both caspase-1 and caspase-8 were present.

(E) LDH release cell death assays of iBMDMs of the indicated genotypes that had been infected with *Salmonella* SL1344 (MOI = 50). Data pooled from two or more experiments. Mean and SEM are shown. ^{ns}p > 0.05 = not significant.

(F) LDH release cell death assays of iBMDMs of the indicated genotypes that had been infected with *Salmonella* SL1344 (MOI = 50). Data pooled from two or more experiments. Mean and SEM are shown. ^{ns}p > 0.05 = not significant.

Please see also Figure S5.

CellPress
OPEN ACCESS

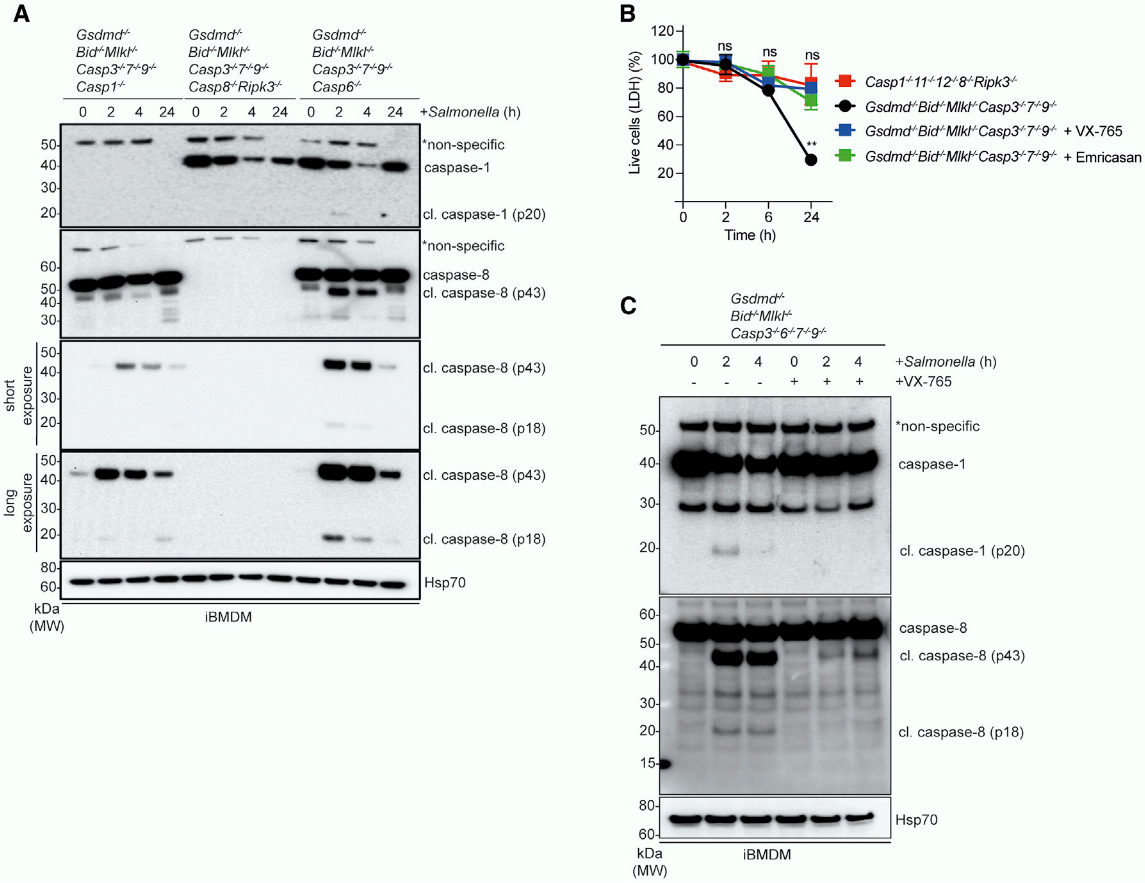

Figure 7. Caspase-1 Can Act Upstream of and Requires Caspase-8 to Induce Cell Death in the Absence of All Known Downstream Effectors of Pyroptosis and Apoptosis

(A) iBMDMs of the indicated genotypes were infected with *Samonella* SL1344 (MOI = 50) and cleavage associated with activation of caspases-1 and -8 was analyzed by immunoblotting at the indicated time points. Probing for HSP70 served as loading control.

(B) LDH release death assays of *Salmonella* SL1344 (MOI = 50) -infected *Casp1*$^{-/-}$;*Casp11*$^{-/-}$;*Casp12*$^{-/-}$;*Casp8*$^{-/-}$;*Ripk3*$^{-/-}$ and *GsdmD*$^{-/-}$;*Bid*$^{-/-}$;*Mlkl*$^{-/-}$;*Casp3*$^{-/-}$;*Casp7*$^{-/-}$;*Casp9*$^{-/-}$ iBMDMs that had been left untreated or treated with VX-765 or Emricasan. Data pooled from two experiments. Mean and SEM are shown. **$p < 0.005$, $^{ns}p > 0.05$ = not significant.

(C) Immunoblot analysis of caspases-1 and -8 activation at the indicated time points in *Salmonella* SL1344 (MOI = 50) -infected *GsdmD*$^{-/-}$;*Bid*$^{-/-}$;*Mlkl*$^{-/-}$;*Casp3*$^{-/-}$;*7*$^{-/-}$;*9*$^{-/-}$ iBMDMs that had been left untreated or treated with VX-765. Probing for HSP70 served as a loading control.

Please see also Figure S6.

Caspase-1 Can Act Upstream of and Requires Caspase-8 to Induce Cell Death in the Absence of All Known Downstream Effectors of Cell Death

We found that the caspase-8 cleavage we had observed in *Salmonella*-infected *Gsdmd*$^{-/-}$;*Bid*$^{-/-}$;*Mlkl*$^{-/-}$;*Casp3*$^{-/-}$;*Casp6*$^{-/-}$;*Casp7*$^{-/-}$;*Casp9*$^{-/-}$ iBMDMs was strongly reduced by the additional deletion of caspase-1 (Figure 7A), indicating that caspase-1 was required for full activation of caspase-8. This identified caspase-1 as the most potent upstream initiator of *Salmonella*-induced cell killing and explained why we only enriched for sgRNAs targeting caspase-1 and its activator NLRC4 in the CRISPR screen. Supporting this idea, we found that both the broad-spectrum caspase inhibitor Emricasan and the highly specific caspase-1 inhibitor VX-765 completely blocked *Salmonella*-induced killing of *Gsdmd*$^{-/-}$;*Bid*$^{-/-}$;*Mlkl*$^{-/-}$;*Casp3*$^{-/-}$;*Casp7*$^{-/-}$;*Casp9*$^{-/-}$ iBMDMs (Figure 7B). Furthermore, similar to the genetic deletion of *Casp1*, VX-765 almost completely blocked caspase-8 processing in *Salmonella*-infected *Gsdmd*$^{-/-}$;*Bid*$^{-/-}$;*Mlkl*$^{-/-}$;*Casp3*$^{-/-}$;*Casp6*$^{-/-}$;*Casp7*$^{-/-}$;*Casp9*$^{-/-}$ cells (Figure 7C). Of note, this type of cell death, which was ensured as long as both caspase-1 and caspase-8 were present, exhibited apoptosis-like morphology as demonstrated by brightfield microscopy of *Salmonella*-infected *Gsdmd*$^{-/-}$;*Bid*$^{-/-}$;*Mlkl*$^{-/-}$;*Casp3*$^{-/-}$;*Casp7*$^{-/-}$;*Casp9*$^{-/-}$ iBMDMs (Figure S6A). These results show that *Salmonella*-infected macrophages can undergo programmed cell death in the absence of all known effector mechanisms of pyroptosis, apoptosis, and necroptosis as long as caspases-1 and -8 can be activated (see schematic shown in Figure S6B).

DISCUSSION

The clearance of intracellular pathogens requires programmed death of the infected cells. However, the relative requirement

for individual cell death pathways and how they are connected at the molecular level were not clear. Our findings uncovered a highly flexible system of cell-death-inducing pathways through which phagocytes can purge bacteria from intracellular niches and thereby enable the host to control intracellular bacteria. While we have observed and functionally validated the triggering of the known pathways leading to pyroptosis, necroptosis, and apoptosis, our work reveals a considerable plasticity that allows bacterially infected cells to rewire known cell death signaling cascades in highly flexible and thus far unknown ways. We identified caspase-1 and caspase-8 as central pillars of this system and demonstrated that multiple previously unknown versions of rewired cell death circuits can efficiently evict bacteria from intracellular, most likely vacuolar niches, as long as one of these central hubs is present.

Our results revealed that caspases-1 and -8, but not -11, could kill *Salmonella*-infected cells in the absence of all known effectors of cell killing (i.e., caspases-3, -6, and -7, BID, and GSDMD). We observed enhanced cleavage of caspase-8 in *Salmonella*-infected iBMDMs that lacked caspase-1. This suggests that the strengths of caspase-8 activation may be subject to regulation by the activity of caspase-1. Our data exclude non-redundant roles for ripoptosomes in mediating interactions between these two central regulators of programed cell death, and it is likely that ASC provided the molecular link between caspases-1 and -8 as previously shown in certain scenarios (Antonopoulos et al., 2015; Lee et al., 2018; Mascarenhas et al., 2017; Pierini et al., 2012; Rauch et al., 2017; Sagulenko et al., 2013; Schneider et al., 2017; Van Opdenbosch et al., 2017). It is possible that the strong activation of caspase-8 in the absence of caspase-1 is indicative of some bona fide, yet-to-be-uncovered inhibitory effects reminiscent of the role of caspase-8 in preventing necroptosis (Oberst et al., 2011). Alternatively, this could reflect differential kinetics whereby the more rapid induction of pyroptosis may simply kill cells before caspase-8 is fully activated, and the role of caspase-8 would thus only become appreciable in the absence of caspase-1. Remarkably, this requirement for caspase-8 downstream of caspase-1 was not absolute, as *Salmonella*-infected iBMDMs lacking caspase-8 and RIPK3 still died as long as caspase-9 was present, even when the effector caspases-3 and -7 were also missing. It is tempting to speculate that caspase-9 was directly activated by caspase-1 through a MOMP-independent mechanism under these conditions, as cell death still depended on caspase-1. Regardless of the precise molecular mechanisms underpinning the observed phenomena, our findings highlight a role for caspase-1 as a master regulator in the orchestration of multiple cell death pathways during infection with intracellular pathogens. This bears resemblance to recent data proposing a similar role for caspase-8 during embryonic development (Fritsch et al., 2019; Newton et al., 2019). Importantly, host defense can also be maintained in the absence of caspase-1, but this depends on the intactness of the downstream effector machinery that, under such conditions, is coordinated by caspase-8 instead.

The various alternative circuits of cell death uncovered here all resulted in morphological features characteristic of apoptosis. Thus, rather than one type of lytic cell death, such as pyroptosis, being compensated for by another form of programmed cellular lysis (i.e., necroptosis), our study and work by others (Mascarenhas et al., 2017; Van Opdenbosch et al., 2017) indicate that the absence of lytic cell death appears to be backed up by apoptosis. This is noteworthy because lytic and non-lytic types of cell death are believed to differ substantially in their consequences for the host, with the former promoting inflammation while the latter is often referred to as immunologically silent. Yet, we found here that *in vivo* control of *Salmonella* proceeded normally, irrespective of these functional differences. More work is required to dissect how the qualitatively different forms of cell death impact on the ultimate clearance of the bacteria through adaptive immune responses mediated by $CD4^+$ T cells and $CD8^+$ T cells (Kupz et al., 2012, 2014).

A potential backup role for caspase-11 in synergizing, enhancing, or even compensating for caspase-1 in host defense against intracellular pathogens has been the focus of previous studies (Broz et al., 2012; Ng and Monack, 2013). Our findings indicate that caspase-11 can indeed play a role (albeit relatively minor) in pathogen clearance, operating independently of caspases-1 and -8. However, caspase-11-mediated killing of *Salmonella*-infected cells was strictly dependent on the activation of GSDMD, demonstrating its limited backup ability compared to caspase-1, which can also kill through caspases-3, -7, -9, and -8 or act as a cell death executioner itself.

Collectively, our work demonstrates substantial flexibility and plasticity with which macrophages can commit suicide to purge *Salmonella* from intracellular hideouts. As long as caspase-1 or caspase-8 can be activated, molecular components previously thought to be unique to particular types of programmed cell death can be flexibly deployed and thereby ensure the killing of *Salmonella*-infected macrophages even when all currently known executioners are absent. Such a complex system has likely arisen as a consequence of host-pathogen co-evolution and the never-ending struggle between pathogens seeking to evade cell death and the host offsetting these attempts through the rewiring of cell death circuits. While we focused on a prototypical intracellular pathogen with global relevance, it is interesting to note that extracellular bacterial pathogens, such as *Staphylococcus aureus*, also express and inject effector molecules capable of manipulating apoptosis into host cells through their type VII secretion systems (Korea et al., 2014; Winstel et al., 2019). This may suggest that programed cell death could also play a role in the host response against extracellular bacteria, and it is tempting to speculate that the extracellular lifestyle of some bacteria could be considered as yet another evasion strategy of the intra-cellular suicide machinery.

STAR★METHODS

Detailed methods are provided in the online version of this paper and include the following:

- KEY RESOURCES TABLE
- RESOURCE AVAILABILITY
  - Lead Contact
  - Materials Availability
  - Data and Code Availability
- EXPERIMENTAL MODEL AND SUBJECT DETAILS
  - Mice
  - Bone Marrow Chimeras

○ Bone Marrow-Derived Macrophages (BMDMs)
○ Immortalized Bone Marrow-Derived Macrophages (iBMDMs)
○ Bacterial Strains for *in vivo* and *in vitro* Infection Studies
● METHOD DETAILS
○ Cell Culture
○ Lentiviral Infection and CRISPR/Cas9 Mediated Gene Deletion
○ LDH Release Cell Death Assay
○ Immunoblotting
○ Immunoprecipitation of Activated BAX
○ Treatment of Cells *in vitro* with BH3 Mimetic Drugs or Etoposide
○ Treatment of Cells *in vitro* with Caspase Inhibitors
○ Treatment of Cells *in vitro* with LPS
○ *Treatment of Cells in vitro with the RIPK1 Inhibitor, Nec1s, and/or TNF-α plus Birinapant and Emricasan*
○ Brightfield, Confocal and Lattice Light Sheet Microscopy
○ CRISPR/Cas9 Whole Genome Guide RNA Library Screen
● QUANTIFICATION AND STATISTICAL ANALYSIS

SUPPLEMENTAL INFORMATION

Supplemental Information can be found online at https://doi.org/10.1016/j.immuni.2020.07.004.

ACKNOWLEDGMENTS

We thank M. Patsis and G. Siciliano for expert animal care, B. Helbert and K. Mackwell for genotyping, S. Monard and his team for help with flow cytometry, V. Dixit and N. Kayagaki for insightful discussions, and all the members of the Herold, Strasser, and Bedoui laboratories. We thank Gordon Dougan (University of Cambridge) for the SL1344 GFP-expressing *Salmonella* strain. This work was supported by grants and fellowships from the Australian National Health and Medical Research Council (NHMRC) (project grants 1186575 and 1145728 to M.J.H., 1143105 to M.J.H. and A.S., 1159658 to M.J.H. and S.B., and 1016701 to A.S.; and fellowships 1020363 to A.S. and 1156095 to M.J.H.), the Leukemia and Lymphoma Society of America (LLS SCOR 7001-13 to A.S. and M.J.H.), the Cancer Council of Victoria (project grants 1147328 to M.J.H. and 1052309 to A.S., and a venture grant to M.J.H. and A.S.), the Australian Phenomics Network (to M.J.H. and A.J.K.), the Cass Foundation (to M.D.), the Wellcome Trust (investigator award 108045/Z/15/Z to C.E.B.), and the German Research Council (GRK2168 to S.E. and S.B.), as well as by operational infrastructure grants through the Australian Government Independent Research Institute Infrastructure Support Scheme (361646 and 9000220) and the Victorian State Government Operational Infrastructure Support Program. The graphical abstract was created with the help of BioRender.com.

AUTHOR CONTRIBUTIONS

M.J.H., A.S., and S.B. conceived the study; and M.J.H., A.S., S.B., and M.D. wrote the manuscript with input from all other authors. M.D. designed and performed experiments and generated the figures; Y.D. generated the CRISPR iBMDM lines and contributed to experiments; P.W., S.E., A.B., E.G., S.W., N.W., C.Y., M. Pereira, R.A.S., and G.E. performed and analyzed experiments; R.S. performed *in vivo* and *in vitro* experiments; N.D.G. and S.M.B. performed and K.L.R. supervised microscopy; A.J.K. and L.T. generated CRISPR reagents; A.L.G. performed the bioinformatics; M.A.D. performed the BAX activation assay; D.D.N. provided reagents; and J.S.P., C.E.B., R.A.S., J.E.V., and M. Pellegrini contributed to experimental design.

DECLARATION OF INTERESTS

The authors declare no competing interests.

Received: March 2, 2020
Revised: June 12, 2020
Accepted: July 2, 2020
Published: July 30, 2020

REFERENCES

Alvarez-Diaz, S., Dillon, C.P., Lalaoui, N., Tanzer, M.C., Rodriguez, D.A., Lin, A., Lebois, M., Hakem, R., Josefsson, E.C., O'Reilly, L.A., et al. (2016). The Pseudokinase MLKL and the Kinase RIPK3 Have Distinct Roles in Autoimmune Disease Caused by Loss of Death-Receptor-Induced Apoptosis. Immunity *45*, 513–526.

Antonopoulos, C., Russo, H.M., El Sanadi, C., Martin, B.N., Li, X., Kaiser, W.J., Mocarski, E.S., and Dubyak, G.R. (2015). Caspase-8 as an Effector and Regulator of NLRP3 Inflammasome Signaling. J. Biol. Chem. *290*, 20167–20184.

Aubrey, B.J., Kelly, G.L., Kueh, A.J., Brennan, M.S., O'Connor, L., Milla, L., Wilcox, S., Tai, L., Strasser, A., and Herold, M.J. (2015). An inducible lentiviral guide RNA platform enables the identification of tumor-essential genes and tumor-promoting mutations in vivo. Cell Rep. *10*, 1422–1432.

Bachem, A., Makhlouf, C., Binger, K.J., de Souza, D.P., Tull, D., Hochheiser, K., Whitney, P.G., Fernandez-Ruiz, D., Dahling, S., Kastenmuller, W., et al. (2019). Microbiota-Derived Short-Chain Fatty Acids Promote the Memory Potential of Antigen-Activated CD8(+) T Cells. Immunity *51*, 285–297.e5.

Bedoui, S., Kupz, A., Wijburg, O.L., Walduck, A.K., Rescigno, M., and Strugnell, R.A. (2010). Different bacterial pathogens, different strategies, yet the aim is the same: evasion of intestinal dendritic cell recognition. J. Immunol. *184*, 2237–2242.

Benoun, J.M., Peres, N.G., Wang, N., Pham, O.H., Rudisill, V.L., Fogassy, Z.N., Whitney, P.G., Fernandez-Ruiz, D., Gebhardt, T., Pham, Q.M., et al. (2018). Optimal protection against *Salmonella* infection requires noncirculating memory. Proc. Natl. Acad. Sci. USA *115*, 10416–10421.

Browne, A.J., Kashef Hamadani, B.H., Kumaran, E.A.P., Rao, P., Longbottom, J., Harriss, E., Moore, C.E., Dunachie, S., Basnyat, B., Baker, S., et al. (2020). Drug-resistant enteric fever worldwide, 1990 to 2018: a systematic review and meta-analysis. BMC Med. *18*, 1.

Broz, P., Newton, K., Lamkanfi, M., Mariathasan, S., Dixit, V.M., and Monack, D.M. (2010). Redundant roles for inflammasome receptors NLRP3 and NLRC4 in host defense against Salmonella. J. Exp. Med. *207*, 1745–1755.

Broz, P., Ruby, T., Belhocine, K., Bouley, D.M., Kayagaki, N., Dixit, V.M., and Monack, D.M. (2012). Caspase-11 increases susceptibility to Salmonella infection in the absence of caspase-1. Nature *490*, 288–291.

Chen, B.C., Legant, W.R., Wang, K., Shao, L., Milkie, D.E., Davidson, M.W., Janetopoulos, C., Wu, X.S., Hammer, J.A., 3rd, Liu, Z., et al. (2014). Lattice light-sheet microscopy: imaging molecules to embryos at high spatiotemporal resolution. Science *346*, 1257998.

Czabotar, P.E., Lessene, G., Strasser, A., and Adams, J.M. (2014). Control of apoptosis by the BCL-2 protein family: implications for physiology and therapy. Nat. Rev. Mol. Cell Biol. *15*, 49–63.

De Nardo, D., Kalvakolanu, D.V., and Latz, E. (2018). Immortalization of Murine Bone Marrow-Derived Macrophages. Methods Mol. Biol. *1784*, 35–49.

Dengler, M.A., Robin, A.Y., Gibson, L., Li, M.X., Sandow, J.J., Iyer, S., Webb, A.I., Westphal, D., Dewson, G., and Adams, J.M. (2019). BAX Activation: Mutations Near Its Proposed Non-canonical BH3 Binding Site Reveal Allosteric Changes Controlling Mitochondrial Association. Cell Rep. *27*, 359–373.e6.

Franchi, L., Amer, A., Body-Malapel, M., Kanneganti, T.D., Ozören, N., Jagirdar, R., Inohara, N., Vandenabeele, P., Bertin, J., Coyle, A., et al. (2006). Cytosolic flagellin requires Ipaf for activation of caspase-1 and interleukin 1beta in salmonella-infected macrophages. Nat. Immunol. *7*, 576–582.

Franchi, L., Eigenbrod, T., Muñoz-Planillo, R., and Nuñez, G. (2009). The inflammasome: a caspase-1-activation platform that regulates immune responses and disease pathogenesis. Nat. Immunol. *10*, 241–247.

Fritsch, M., Günther, S.D., Schwarzer, R., Albert, M.C., Schorn, F., Werthenbach, J.P., Schiffmann, L.M., Stair, N., Stocks, H., Seeger, J.M., et al. (2019). Caspase-8 is the molecular switch for apoptosis, necroptosis and pyroptosis. Nature *575*, 683–687.

Green, D.R. (2019). The Coming Decade of Cell Death Research: Five Riddles. Cell *177*, 1094–1107.

Hautefort, I., Proença, M.J., and Hinton, J.C. (2003). Single-copy green fluorescent protein gene fusions allow accurate measurement of Salmonella gene expression in vitro and during infection of mammalian cells. Appl. Environ. Microbiol. *69*, 7480–7491.

Heilig, R., Dilucca, M., Boucher, D., Chen, K.W., Hancz, D., Demarco, B., Shkarina, K., and Broz, P. (2020). Caspase-1 cleaves Bid to release mitochondrial SMAC and drive secondary necrosis in the absence of GSDMD. Life Sci. Alliance *3*, https://doi.org/10.26508/lsa.202000735.

Jorgensen, I., Rayamajhi, M., and Miao, E.A. (2017). Programmed cell death as a defence against infection. Nat. Rev. Immunol. *17*, 151–164.

Kaiser, W.J., Upton, J.W., Long, A.B., Livingston-Rosanoff, D., Daley-Bauer, L.P., Hakem, R., Caspary, T., and Mocarski, E.S. (2011). RIP3 mediates the embryonic lethality of caspase-8-deficient mice. Nature *471*, 368–372.

Kayagaki, N., Stowe, I.B., Lee, B.L., O'Rourke, K., Anderson, K., Warming, S., Cuellar, T., Haley, B., Roose-Girma, M., Phung, Q.T., et al. (2015). Caspase-11 cleaves gasdermin D for non-canonical inflammasome signalling. Nature *526*, 666–671.

Koike-Yusa, H., Li, Y., Tan, E.P., Velasco-Herrera, Mdel.C., and Yusa, K. (2014). Genome-wide recessive genetic screening in mammalian cells with a lentiviral CRISPR-guide RNA library. Nat. Biotechnol. *32*, 267–273.

Korea, C.G., Balsamo, G., Pezzicoli, A., Merakou, C., Tavarini, S., Bagnoli, F., Serruto, D., and Unnikrishnan, M. (2014). Staphylococcal Esx proteins modulate apoptosis and release of intracellular Staphylococcus aureus during infection in epithelial cells. Infect. Immun. *82*, 4144–4153.

Kueh, A.J., and Herold, M.J. (2016). Using CRISPR/Cas9 Technology for Manipulating Cell Death Regulators. Methods Mol. Biol. *1419*, 253–264.

Kuida, K., Lippke, J.A., Ku, G., Harding, M.W., Livingston, D.J., Su, M.S.-S., and Flavell, R.A. (1995). Altered cytokine export and apoptosis in mice deficient in interleukin-1 beta converting enzyme. Science *267*, 2000–2003.

Kupz, A., Guarda, G., Gebhardt, T., Sander, L.E., Short, K.R., Diavatopoulos, D.A., Wijburg, O.L., Cao, H., Waithman, J.C., Chen, W., et al. (2012). NLRC4 inflammasomes in dendritic cells regulate noncognate effector function by memory $CD8^+$ T cells. Nat. Immunol. *13*, 162–169.

Kupz, A., Scott, T.A., Belz, G.T., Andrews, D.M., Greyer, M., Lew, A.M., Brooks, A.G., Smyth, M.J., Curtiss, R., 3rd, Bedoui, S., and Strugnell, R.A. (2013). Contribution of Thy1+ NK cells to protective IFN-γ production during Salmonella typhimurium infections. Proc. Natl. Acad. Sci. USA *110*, 2252–2257.

Kupz, A., Bedoui, S., and Strugnell, R.A. (2014). Cellular requirements for systemic control of Salmonella enterica serovar Typhimurium infections in mice. Infect. Immun. *82*, 4997–5004.

Lamkanfi, M., and Dixit, V.M. (2014). Mechanisms and functions of inflammasomes. Cell *157*, 1013–1022.

Lee, B.L., Mirrashidi, K.M., Stowe, I.B., Kummerfeld, S.K., Watanabe, C., Haley, B., Cuellar, T.L., Reichelt, M., and Kayagaki, N. (2018). ASC- and caspase-8-dependent apoptotic pathway diverges from the NLRC4 inflammasome in macrophages. Sci. Rep. *8*, 3788.

Man, S.M., Karki, R., Briard, B., Burton, A., Gingras, S., Pelletier, S., and Kanneganti, T.D. (2017). Differential roles of caspase-1 and caspase-11 in infection and inflammation. Sci. Rep. *7*, 45126.

Mariathasan, S., Newton, K., Monack, D.M., Vucic, D., French, D.M., Lee, W.P., Roose-Girma, M., Erickson, S., and Dixit, V.M. (2004). Differential activation of the inflammasome by caspase-1 adaptors ASC and Ipaf. Nature *430*, 213–218.

Mascarenhas, D.P.A., Cerqueira, D.M., Pereira, M.S.F., Castanheira, F.V.S., Fernandes, T.D., Manin, G.Z., Cunha, L.D., and Zamboni, D.S. (2017). Inhibition of caspase-1 or gasdermin-D enable caspase-8 activation in the Naip5/NLRC4/ASC inflammasome. PLoS Pathog. *13*, e1006502.

Miao, E.A., Leaf, I.A., Treuting, P.M., Mao, D.P., Dors, M., Sarkar, A., Warren, S.E., Wewers, M.D., and Aderem, A. (2010). Caspase-1-induced pyroptosis is an innate immune effector mechanism against intracellular bacteria. Nat. Immunol. *11*, 1136–1142.

Murphy, J.M., Czabotar, P.E., Hildebrand, J.M., Lucet, I.S., Zhang, J.G., Alvarez-Diaz, S., Lewis, R., Lalaoui, N., Metcalf, D., Webb, A.I., et al. (2013). The pseudokinase MLKL mediates necroptosis via a molecular switch mechanism. Immunity *39*, 443–453.

Nagata, S. (2018). Apoptosis and Clearance of Apoptotic Cells. Annu. Rev. Immunol. *36*, 489–517.

Newton, K., Wickliffe, K.E., Maltzman, A., Dugger, D.L., Reja, R., Zhang, Y., Roose-Girma, M., Modrusan, Z., Sagolla, M.S., Webster, J.D., and Dixit, V.M. (2019). Activity of caspase-8 determines plasticity between cell death pathways. Nature *575*, 679–682.

Ng, T.M., and Monack, D.M. (2013). Revisiting caspase-11 function in host defense. Cell Host Microbe *14*, 9–14.

Oberst, A., Dillon, C.P., Weinlich, R., McCormick, L.L., Fitzgerald, P., Pop, C., Hakem, R., Salvesen, G.S., and Green, D.R. (2011). Catalytic activity of the caspase-8-FLIP(L) complex inhibits RIPK3-dependent necrosis. Nature *471*, 363–367.

Ofengeim, D., and Yuan, J. (2013). Regulation of RIP1 kinase signalling at the crossroads of inflammation and cell death. Nat. Rev. Mol. Cell Biol. *14*, 727–736.

Orning, P., Weng, D., Starheim, K., Ratner, D., Best, Z., Lee, B., Brooks, A., Xia, S., Wu, H., Kelliher, M.A., et al. (2018). Pathogen blockade of TAK1 triggers caspase-8-dependent cleavage of gasdermin D and cell death. Science *362*, 1064–1069.

Pierini, R., Juruj, C., Perret, M., Jones, C.L., Mangeot, P., Weiss, D.S., and Henry, T. (2012). AIM2/ASC triggers caspase-8-dependent apoptosis in Francisella-infected caspase-1-deficient macrophages. Cell Death Differ. *19*, 1709–1721.

Rauch, I., Deets, K.A., Ji, D.X., von Moltke, J., Tenthorey, J.L., Lee, A.Y., Philip, N.H., Ayres, J.S., Brodsky, I.E., Gronert, K., and Vance, R.E. (2017). NAIP-NLRC4 Inflammasomes Coordinate Intestinal Epithelial Cell Expulsion with Eicosanoid and IL-18 Release via Activation of Caspase-1 and -8. Immunity *46*, 649–659.

Robinson, M.D., and Oshlack, A. (2010). A scaling normalization method for differential expression analysis of RNA-seq data. Genome Biol. *11*, R25.

Robinson, M.D., McCarthy, D.J., and Smyth, G.K. (2010). edgeR: a Bioconductor package for differential expression analysis of digital gene expression data. Bioinformatics *26*, 139–140.

Sagulenko, V., Thygesen, S.J., Sester, D.P., Idris, A., Cridland, J.A., Vajjhala, P.R., Roberts, T.L., Schroder, K., Vince, J.E., Hill, J.M., et al. (2013). AIM2 and NLRP3 inflammasomes activate both apoptotic and pyroptotic death pathways via ASC. Cell Death Differ. *20*, 1149–1160.

Salvamoser, R., Brinkmann, K., O'Reilly, L.A., Whitehead, L., Strasser, A., and Herold, M.J. (2019). Characterisation of mice lacking the inflammatory caspases-1/11/12 reveals no contribution of caspase-12 to cell death and sepsis. Cell Death Differ. *26*, 1124–1137.

Salvesen, G.S., and Dixit, V.M. (1997). Caspases: intracellular signaling by proteolysis. Cell *91*, 443–446.

Schneider, K.S., Groß, C.J., Dreier, R.F., Saller, B.S., Mishra, R., Gorka, O., Heilig, R., Meunier, E., Dick, M.S., Ćiković, T., et al. (2017). The Inflammasome Drives GSDMD-Independent Secondary Pyroptosis and IL-1 Release in the Absence of Caspase-1 Protease Activity. Cell Rep. *21*, 3846–3859.

Shi, J., Zhao, Y., Wang, K., Shi, X., Wang, Y., Huang, H., Zhuang, Y., Cai, T., Wang, F., and Shao, F. (2015). Cleavage of GSDMD by inflammatory caspases determines pyroptotic cell death. Nature *526*, 660–665.

CellPress
OPEN ACCESS

Tenev, T., Bianchi, K., Darding, M., Broemer, M., Langlais, C., Wallberg, F., Zachariou, A., Lopez, J., MacFarlane, M., Cain, K., and Meier, P. (2011). The Ripoptosome, a signaling platform that assembles in response to genotoxic stress and loss of IAPs. Mol. Cell *43*, 432–448.

Tsuchiya, K., Nakajima, S., Hosojima, S., Thi Nguyen, D., Hattori, T., Manh Le, T., Hori, O., Mahib, M.R., Yamaguchi, Y., Miura, M., et al. (2019). Caspase-1 initiates apoptosis in the absence of gasdermin D. Nat. Commun. *10*, 2091.

Van Opdenbosch, N., Van Gorp, H., Verdonckt, M., Saavedra, P.H.V., de Vasconcelos, N.M., Gonçalves, A., Vande Walle, L., Demon, D., Matusiak, M., Van Hauwermeiren, F., et al. (2017). Caspase-1 Engagement and TLR-Induced c-FLIP Expression Suppress ASC/Caspase-8-Dependent Apoptosis by Inflammasome Sensors NLRP1b and NLRC4. Cell Rep. *21*, 3427–3444.

Vandenabeele, P., Galluzzi, L., Vanden Berghe, T., and Kroemer, G. (2010). Molecular mechanisms of necroptosis: an ordered cellular explosion. Nat. Rev. Mol. Cell Biol. *11*, 700–714.

Winstel, V., Schneewind, O., and Missiakas, D. (2019). Staphylococcus aureus Exploits the Host Apoptotic Pathway To Persist during Infection. MBio *10*, https://doi.org/10.1128/mBio.02270-19.

Zhang, L., Chen, S., Ruan, J., Wu, J., Tong, A.B., Yin, Q., Li, Y., David, L., Lu, A., Wang, W.L., et al. (2015). Cryo-EM structure of the activated NAIP2-NLRC4 inflammasome reveals nucleated polymerization. Science *350*, 404–409.

Zhao, Y., and Shao, F. (2016). Diverse mechanisms for inflammasome sensing of cytosolic bacteria and bacterial virulence. Curr. Opin. Microbiol. *29*, 37–42.

STAR★METHODS

KEY RESOURCES TABLE

REAGENT or RESOURCE	SOURCE	IDENTIFIER
Antibodies		
rat anti-caspase-11 (4E11)	Enzo Life Sciences	Cat# ALX-804-530-C100; RRID: AB_2050921
rat anti-caspase-1 (1H11)	Enzo Life Sciences	Cat# ALX-804-507-C100; RRID: AB_2050924
rat anti-BID (2D1-3)	WEHI	N/A
mouse anti-PARP (C2-10)	Santa Cruz	Cat# sc-53643; RRID: AB_785086
rabbit anti-GSDMD	Abcam	Cat# EPR19828; RRID: AB_2783550
rabbit anti-Bak	Sigma Aldrich	Cat# B-5897; RRID: AB_258581
rat anti-Bax (5B7)	SouthernBiotech	Cat# 10050-01; RRID: AB_2794106
Rabbit polyclonal anti-BAX NT	Merck Millipore	Cat#ABC11; RRID: AB_310143
anti-BAX antibody 6A7 (aa113-19)	BD Biosciences	Cat# 556467; RRID: AB_396430
rabbit anti-caspase-9	CST	Cat# 9504; RRID: AB_2275591
rabbit anti-caspase-7 (D2Q3L)	CST	Cat# 12827S; RRID: AB_2687912
rabbit anti-cleaved caspase-3 (Asp175)	CST	Cat# 9661S; RRID: AB_2341188
rabbit anti-RIPK3	ProSci	Cat# 2283; RRID: AB_203256
rabbit anti-cleaved caspase-8 (D5B2)	CST	Cat# 8592S; RRID: AB_10891784
rat anti-caspase-8 (3B10)	Enzo Life Sciences	Cat# ALX-804-448-C100; RRID: AB_2050953
rabbit anti-phospho MLKL (S345)	Abcam	Cat# ab196436; RRID: AB_2687465
rat anti-MLKL (3H1)	Merck	Cat# MABC604; RRID: AB_2820284
anti-β-actin-HRP (13E5)	CST	Cat# 5125S; RRID: AB_1903890
mouse anti-HSP70 (BRM-22)	Sigma Aldrich	Cat# MA1-91159; RRID: AB_1957733
Goat anti-rabbit Ig (H/L): HRP conjugate	Southern Biotech	Cat# 4010-05; RRID: AB_2632593
Goat anti-rat Ig (H/L): HRP conjugate	Southern Biotech	Cat# 3010-05; RRID: AB_619911
Goat anti-mouse Ig (H/L): HRP conjugate	Southern Biotech	Cat# 1010-05; RRID: AB_609673
Goat anti-rabbit Ig (Fc): HRP conjugate	Southern Biotech	Cat# 3030-05; RRID: AB_2716837
Bacterial and Virus Strains		
Stbl3 chemically competent *E. coli*	Invitrogen	Cat# C737303
Chemicals, Peptides, and Recombinant Proteins		
Gentamycin	Sigma Aldrich	Cat# G-1397
ABT-199 (Venetoclax) (BCL-2i)	ActiveBiochem	Cat# A-1231
S63845 (MCL-1i)	ActiveBiochem	Cat# A-6044
A1331852 (BCL-XLi)	ActiveBiochem	Cat# A-6046
Etoposide	Sigma Aldrich	Cat# E-1383
RIP1 inhibitor II, 7-Cl⁻O-Nec (10mg) (Nec1s)	Merck	Cat# 5.04297.0001
TNF-α	Miltenyi	Cat# 130-101-690
Birinapant	TetraLogic/Medivir	N/A
Emricasan	MedKoo	Cat# 510230
VX-765	InvivoGen	Cat# inh-vx765i-1
PhosSTOP phosphatase inhibitor	Roche	Cat# 04906837001
EDTA-free Protease inhibitor cocktail	Roche	Cat# 11836170001
Proteinase K	Roche	Cat# 3115879
dox hyclate	Sigma-Aldrich	Cat# D-9891
Luminata Forte Western HRP substrate	Merck Millipore	Cat# WBLUF0500
Lipopolysaccharide from *E.coli*	Sigma-Aldrich	Cat# L-2880
Critical Commercial Assays		
Promega CyTox LDH assay	Promega	Cat# G1780

(*Continued on next page*)

Continued

REAGENT or RESOURCE	SOURCE	IDENTIFIER
Experimental Models: Cell Lines		
Primary murine BMDMs	this manuscript	N/A
murine WT iBMDMs	(De Nardo et al., 2018)	N/A
murine *Casp1/11/12*^{KO} iBMDMs	this manuscript	N/A
murine *Casp1/11/12/8/RipK3*^{KO} iBMDMs	this manuscript	N/A
murine *Casp8/RipK3*^{KO} iBMDMs	this manuscript	N/A
murine *MLKL*^{KO} iBMDMs	this manuscript	N/A
murine *Casp1/8/RipK3*^{KO} iBMDMs	this manuscript	N/A
murine *Casp1/11/8/RipK3*^{KO} iBMDMs	this manuscript	N/A
murine *Casp1/8/RipK3/Gsdmd*^{KO} iBMDMs	this manuscript	N/A
murine *Gsdmd*^{KO} iBMDMs	this manuscript	N/A
murine *Gsdmd/Casp8/Ripk3*^{KO} iBMDMs	this manuscript	N/A
murine *Gsdmd/Casp8/Ripk3/Bax/Bak*^{KO} iBMDMs	this manuscript	N/A
murine *Gsdmd/Casp8/Ripk3/Mlkl/Casp3/7*^{KO} iBMDMs	this manuscript	N/A
murine *Gsdmd/Casp8/Ripk3/Mlkl/Casp3/6/7*^{KO} iBMDMs	this manuscript	N/A
murine *Gsdmd/Casp8/Ripk3/Mlkl/Casp3/7/9*^{KO} iBMDMs	this manuscript	N/A
murine *Gsdmd/Bid/Mlkl/Casp3/7/9*^{KO} iBMDMs	this manuscript	N/A
murine *Gsdmd/Bid/Mlkl/Casp3/6/7/9*^{KO} iBMDMs	this manuscript	N/A
murine *Gsdmd/Bid/Mlkl/Casp1/3/7/9*^{KO} iBMDMs	this manuscript	N/A
Experimental Models: Organisms/Strains		
Mice: C57BL/6 (WT)	WEHI	N/A
Mice: *MLKL*$^{-/-}$	(Murphy et al., 2013)	N/A
Mice: *Casp1/11*$^{-/-}$	(Kuida et al., 1995)	N/A
Mice: *Casp1/11/12*$^{-/-}$	(Salvamoser et al., 2019)	N/A
Mice: *Casp1/11/12/Ripk3*$^{-/-}$	this manuscript	N/A
Mice: *Casp1/11/12/8/Ripk3*$^{-/-}$	this manuscript	N/A
Mice: *Casp8/Ripk3*$^{-/-}$	(Oberst et al., 2011)	N/A
Salmonella Typhimurium: SL1344	ATCC	Cat#14028
Salmonella Typhimurium: *ΔAroA*	(Kupz et al., 2014)	N/A
Salmonella Typhimurium: SL1344 SPI2 *ssaG-GFP+*	(Hautefort et al., 2003)	N/A
Recombinant DNA		
pVSVg plasmid	Addgene	Cat# 8454
pMDLg/pRRE plasmid	Addgene	Cat# 12251
pRSV-Rev plasmid	Addgene	Cat# 12253
pFH1tUTG- H1-Tet-sgRNA plasmid	(Aubrey et al., 2015), Addgene	Cat#70183
pFUGW-Cas9mcherry plasmid	(Aubrey et al., 2015), Addgene	Cat#70182
Forward Primers for *Caspase-1* sgRNA: 5′-ACTTGCAAACATTACTGCTA-3′	this manuscript	N/A
Reverse Primers for *Caspase-1* sgRNA: 5′-TAGCAGTAATGTTTGCAAGT-3′	this manuscript	N/A
Forward Primers for *Caspase-3* sgRNA: 5′-ATCTCGCTCTGGTACGGATG-3′	this manuscript	N/A
Reverse Primers for *Caspase-3* sgRNA: 5′-CATCCGTACCAGAGCGAGAT-3′	this manuscript	N/A
Forward Primers for *Caspase-6* sgRNA: 5′-TGGCGTCGTATGCGTAAACG-3′	this manuscript	N/A
Reverse Primers for *Caspase-6* sgRNA: 5′-CGTTTACGCATACGACGCCA-3′	this manuscript	N/A

(*Continued on next page*)

Continued

REAGENT or RESOURCE	SOURCE	IDENTIFIER
Forward Primers for *Caspase-7* sgRNA: 5′-GCCCACTTATCTGTACCGCA-3′	this manuscript	N/A
Reverse Primers for *Caspase-7* sgRNA: 5′-TGCGGTACAGATAAGTGGGC-3′	this manuscript	N/A
Forward Primers for *Caspase-8* sgRNA: 5′-TAGCTTCTGGGCATCCTCGA-3′	this manuscript	N/A
Reverse Primers for *Caspase-8* sgRNA: 5′-TCGAGGATGCCCAGAAGCTA-3′	this manuscript	N/A
Forward Primers for *Caspase-9* sgRNA: 5′-AACTTGAGCACCGATTCCGC-3′	this manuscript	N/A
Reverse Primers for *Caspase-9* sgRNA: 5′-GCGGAATCGGTGCTCAAGTT-3′	this manuscript	N/A
Forward Primers for *Caspase-11* sgRNA: 5′- AGCCTTTCGTGTACGGCCAT −3′	this manuscript	N/A
Reverse Primers for *Caspase-11* sgRNA: 5′- ATGGCCGTACACGAAAGGCT −3′	this manuscript	N/A
Forward Primers for *Caspase-12* sgRNA: 5′-TGCGAGTTTCATCCTGAACA-3′	this manuscript	N/A
Reverse Primers for *Caspase-12* sgRNA: 5′-TGTTCAGGATGAAACTCGCA-3′	this manuscript	N/A
Forward Primers for *Ripk3* sgRNA: 5′-GGAACCGCTGACGCACCAGT-3′	this manuscript	N/A
Reverse Primers for *Ripk3* sgRNA: 5′-ACTGGTGCGTCAGCGGTTCC-3′	this manuscript	N/A
Forward Primers for *Gsdmd* sgRNA: 5′-CAGAGGCGATCTCATTCCGG-3′	this manuscript	N/A
Reverse Primers for *Gsdmd* sgRNA: 5′-CCGGAATGAGATCGCCTCTG-3′	this manuscript	N/A
Forward Primers for *Bid* sgRNA: 5′-GGTCAGCAACGGTTCCGGCC-3′	this manuscript	N/A
Reverse Primers for *Bid* sgRNA: 5′-GGCCGGAACCGTTGCTGACC-3′	this manuscript	N/A
Forward Primers for *Mlkl* sgRNA: 5′-TACCCAACACTTTCGGCCTG-3′	this manuscript	N/A
Reverse Primers for *Mlkl* sgRNA: 5′-CAGGCCGAAAGTGTTGGGTA-3′	this manuscript	N/A
Forward Primers for *Caspase12* indel sequencing: 5′-TTACAGCCAGGAGGACACAT-3′	this manuscript	N/A
Reverse Primers for *Caspase12* indel sequencing: 5′-ACAGTCTAAGGGATATGGGG-3′	this manuscript	N/A
**Software and Algorithms**		
GraphPad Prism	Version 8.0d; GraphPad Software Inc.	https://www.graphpad.com/scientific-software/prism/
Image Lab	Version 6.0.0	Bio-Rad laboratories
Adobe Illustrator CC	Version 2015.1.0	http://www.adobe.com/Illustrator
Fiji	Version 2.0.0-rc-69/1.52v	ImageJ: https://imagej.net/Fiji
IMARIS	Version 9.5	Oxford Instruments - Imaris
edgeR	(Robinson et al., 2010)	N/A
**Other**		
Protein G Sepharose® 4 fastflow	GE Healthcare	Cat#17-0618-01

RESOURCE AVAILABILITY

Lead Contact

Further information and requests for resources and reagents should be directed to and will be fulfilled by the Lead Contact and corresponding author, Andreas Strasser (strasser@wehi.edu.au).

Materials Availability

The mouse lines and iBMDM cell lines generated in this study may be obtained (pending continued availability) from the Lead Contact with a completed Materials Transfer Agreement.

Data and Code Availability

The published article includes all datasets generated or analyzed during this study. The full CRISPR/Cas9 whole genome screen dataset supporting the current study may be obtained from the Lead Contact upon request.

EXPERIMENTAL MODEL AND SUBJECT DETAILS

Mice

C57BL/6 (WT), *Mlkl*$^{-/-}$ (Murphy et al., 2013), *Casp8*$^{-/-}$;*Ripk3*$^{-/-}$ (Oberst et al., 2011), *Casp1*$^{-/-}$;*Casp11*$^{-/-}$ (Kuida et al., 1995), *Casp1*$^{-/-}$;*Casp11*$^{-/-}$;*Casp12*$^{-/-}$ (Salvamoser et al., 2019), *Casp1*$^{-/-}$;*Casp11*$^{-/-}$;*Casp12*$^{-/-}$;*Ripk3*$^{-/-}$, and *Casp1*$^{-/-}$;*Casp11*$^{-/-}$;*Casp12*$^{-/-}$;*Casp8*$^{-/-}$;*Ripk3*$^{-/-}$mice were bred and maintained at The Walter and Eliza Hall Institute of Medical Research Animal Facility. Both age- and sex-matched animals between eight and fourteen weeks of age were used for *in vivo* and *in vitro* studies. All mice were bred and housed in specific pathogen-free facilities, in a 12 h light/dark cycle in ventilated cages, with free access to chow and water supply *ad libitum*. All animal experiments were approved by The Walter and Eliza Hall Institute of Medical Research Animal Ethics Committee and The University of Melbourne Animal Ethics Committee (AEC 1714194) and were conducted in accordance with the Prevention of Cruelty to Animals Act (1986) and the Australian National Health and Medical Research Council Code of Practice for the Care and Use of Animals for Scientific Purposes (1997). Accordingly, mice were euthanized at a weight loss of more than 15%, which is described in here as 'mouse survival'.

Bone Marrow Chimeras

Bone marrow chimeras were generated as previously described (Bachem et al., 2019). C57BL/6-CD45.1 mice were lethally irradiated with 2 doses of 550 rad 4 h apart and reconstituted with 5 × 10^6 T cell-depleted bone marrow cells from *Casp1*$^{-/-}$;*Casp11*$^{-/-}$;*Casp12*$^{-/-}$;*Casp8*$^{-/-}$;*Ripk3*$^{-/-}$ (C57BL/6-CD45.2) mice. Chimeric mice were allowed to reconstitute for at least 8 weeks before use in experiments.

Bone Marrow-Derived Macrophages (BMDMs)

Bone marrow-derived macrophages (BMDMs) were prepared by flushing bone marrow from femurs and tibiae of both male and female mice, and culturing cells in DMEM supplemented with 10% fetal bovine serum (FBS; Sigma-Aldrich), 15% L929-conditioned medium, 100 U/mL penicillin and 100 mg/mL streptomycin for six days in non-tissue culture treated dishes.

Immortalized Bone Marrow-Derived Macrophages (iBMDMs)

C57BL/6 *Cre-J2* immortalized bone marrow-derived macrophages (iBMDM) (De Nardo et al., 2018) were passaged in DMEM supplemented with 10% FBS, 100 U/mL penicillin and 100 mg/mL streptomycin at 37°C and 10% CO_2. CRISPR/Cas9 mediated gene deletion was achieved as previously described (Aubrey et al., 2015; Kueh and Herold, 2016). sgRNAs targeting the genes to be deleted were designed *in silico* and cloned into an inducible lentiviral expression vector. Lentivirus was generated using 293T cells and 1 × 10^5 target iBMDM cells transduced with the respective virus supernatant. Infected cells were expanded and single cell sorted into tissue culture medium containing 1 μg/mL dox hyclate (to induce sgRNA expression) (Sigma-Aldrich D9891) performed on eGFP and mCherry expressing populations using FACSaria flow cytometer. Single cell clones were expanded and gene deletion confirmed by western blot analysis of the targeted protein as described above.

Bacterial Strains for *in vivo* and *in vitro* Infection Studies

For *in vivo* infection, *S.* Typhimurium Δ*AroA* was grown shaking at 37°C in Luria-Bertani (LB) broth supplemented with 50 μg/mL streptomycin for 16 to 18 h, diluted in PBS and 200 CFU were injected into the tail vein in a volume of 200 μL. The number of replicating bacteria was determined by homogenizing organs from infected mice in 5 mL of sterile PBS. The homogenate was serially diluted and plated onto LB agar plates supplemented with 50 μg/mL streptomycin. Plates were incubated at 37°C for 24 h. *S.* Typhimurium strain SL1344 was used for *in vitro* infection of primary BMDMs and iBMDMs. Imaging studies utilized SL1344 expressing GFP under the control of the SPI-2 promoter. SL1344 was grown shaking at 37°C over night in (LB) broth (+50 μg/mL Streptomycin) for 16 to 18 h and OD_{600} was determined using a spectrophotometer to calculate multiplicity of infection (MOI). Cells were infected

with SL1344 at MOI of 50 in serum free and antibiotic free medium. After 1 h, cells were washed twice with warm PBS and medium replaced with serum containing Dulbecco's modified Eagle's medium DMEM with 50 μg/mL Gentamycin to prevent growth of extracellular bacteria.

METHOD DETAILS

Cell Culture

BMDMs were passaged in DMEM supplemented with 10% FBS, 15% L929-conditioned medium, 100 U/mL penicillin and 100 mg/mL streptomycin at 37°C and 10% CO_2. iBMDM were passaged in DMEM supplemented with 10% FBS, 100 U/mL penicillin and 100 mg/mL streptomycin at 37°C and 10% CO_2. For experimental assays, cells were seeded into 6- or 96-well plates at a density of 3×10^5 or 2×10^4 cells/well, respectively, in antibiotic-free medium and rested for 24 h before infection/treatment and downstream analysis as described below.

Lentiviral Infection and CRISPR/Cas9 Mediated Gene Deletion

CRISPR/Cas9 mediated gene deletion was achieved as previously described (Aubrey et al., 2015; Kueh and Herold, 2016). sgRNAs targeting the genes to be deleted were designed *in silico* and cloned into an inducible lentiviral expression vector as previously described (Aubrey et al., 2015). Lentivirus was generated using 293T cells and 1×10^5 target iBMDM cells transduced with the respective virus supernatant. Infected cells were expanded and single cell sorts into tissue culture medium containing 1 μg/mL dox hyclate (to induce sgRNA expression) (Sigma-Aldrich D9891) performed on eGFP and mCherry expressing populations using a FACSaria flow cytometer. Single cell clones were expanded and gene deletion confirmed via immunoblot analysis of the targeted protein as described below or in the case of caspase-12 by Sanger sequencing or NGS as previously described (Aubrey et al., 2015) using primers outlined in the Key resources table.

LDH Release Cell Death Assay

The viability of uninfected, *Salmonella*-infected and/or inhibitor treated BMDMs and iBMDMs at the indicated time points was determined using the CytoTox 96® Non-Radioactive Cytotoxicity Assay (Promega). The percentage of live cells at each time point was calculated comparing LDH release of surviving cells in *Salmonella*-infected wells to LDH release of non-infected control cells.

Immunoblotting

To quantify the amounts and to determine the activation status of a wide range of cell death initiator and effector molecules upon *Salmonella* infection, cells were lysed at the indicated time points by scraping with cell lysis buffer containing 20 mM Tris-HCl, pH 7.5, 135 mM NaCl, 1.5 mM Mg_2Cl, 1 mM EGTA, 1% Triton X-100 (Sigma-Aldrich), 10% glycerol, EDTA-free protease inhibitor tablets (Roche, Basel, Switzerland), and phosphatase inhibitor tablets (Roche). Cell lysates were rotated at 4°C for 20 min and then clarified at 4°C at 13,000 g for 15 min. Absolute protein content of clarified lysates was determined by Bradford assay (Bio-Rad, Hercules, CA, USA), and equal quantities (20–50 μg) of total protein were separated under denaturing and reducing conditions (with 5% β-mercaptoethanol) using 4%–12% SDS-PAGE gels (Life Technologies). Proteins were transferred onto nitrocellulose membranes, blocked with either 5% skim milk (Devondale, Brunswick, Australia) or 5% BSA (for phospho-proteins) in PBS with 0.05% Tween-20 (PBST) for 1 h, and detected using the following primary antibodies: rat anti-caspase-11 (4E11, Enzo Life Sciences), rat anti-caspase-1 (1E11, Enzo Life Sciences), rat anti-MLKL (3H1; available from Merck), rabbit anti-phospho S345 MLKL (EPR9515[2]; Abcam, Cambridge, UK), rat anti-caspase-8 (3B10; Enzo Life Sciences), rabbit anti-cleaved caspase-8 (D5B2; Cell Signaling Technology), rabbit anti-RIPK3 (ProSci, Poway, CA, USA), rabbit anti-cleaved caspase-3 (Asp175; Cell Signaling Technology), rabbit anti-caspase-7 (D2Q3L, Cell Signaling), rabbit anti-caspase-9 (Cell Signaling Technology), rabbit anti-GSDMD (EPR19828, Abcam), mouse anti-PARP (C2-10, Santa Cruz), rat anti-BID (2D1-3, WEHI), mouse anti-HSP70 (BRM-22, Sigma Alrich) and rabbit anti-β-actin-HRP (Cell Signaling Technology).

HRP-conjugated goat antibodies against mouse, rat or rabbit IgG (Southern Biotech, Birmingham, AL, USA) were applied as a secondary reagent to membranes, which were subsequently incubated with Amersham ECL Prime Western Blotting Detection Reagent (GE Healthcare) and imaged using a ChemiDoc Touch Imaging System (Bio-Rad). Densitometry was performed using Image Lab v.5.2.1 software (Bio-Rad).

Immunoprecipitation of Activated BAX

To determine BAX activation, 3×10^5 WT and *Casp1*$^{-/-}$;*Casp11*$^{-/-}$;*Casp12*$^{-/-}$;*Casp8*$^{-/-}$;*Ripk3*$^{-/-}$ primary BMDMs or *Casp1*$^{-/-}$;*Casp11*$^{-/-}$;*Casp12*$^{-/-}$;*Casp8*$^{-/-}$;*Ripk3*$^{-/-}$ iBMDMs were left untreated, infected with *Salmonella* and/or treated with a combination of BH3-mimetic drugs (2 μM of each BCL-2i ABT-199, MCL-1i S63845, BCL-XLi A1331852) for 16 h and cells were then solubilized with 1% CHAPS for 30 min on ice. Lysates were centrifuged at 13,000 g for 5 min and pre-cleared with 25 μL Protein G Sepharose (Amersham Biosciences). Pre-cleared supernatant was then incubated with antibody (4 μg) of 6A7 anti-BAX antibody (aa113-19, BD Biosciences Cat# 556467, RRID: AB_396430) and Protein-G Sepharose for 2 h at 4°C. Unbound proteins were collected and the resin washed with lysis buffer containing up to 0.1% w/v CHAPS. Immunoprecipitated proteins (IP) were eluted by boiling in SDS-containing sample buffer. Immunoprecipitates and pre-IP samples were electrophoresed on SDS-PAGE and immunoblotted for BAX (anti-BAX NT). To avoid signals from immunoglobulin (Ig) light chains in immunoblots, Ig heavy chain-specific HRP-conjugated

goat anti-rabbit IgG antibodies (Southern Biotech Cat# 4041-05) were used as secondary reagent, as previously described (Dengler et al., 2019).

Treatment of Cells *in vitro* with BH3 Mimetic Drugs or Etoposide

Primary BMDMs or iBMDMs were seeded for LDH assay or BAX activation analysis as indicated above. The following BH3 mimetic drugs were used for the indicated time points at a final concentration of 2 μM: MCL-1i S63845, BCL-2i ABT199, BCL-XLi A1331852. Etoposide (Sigma) was used at a final concentration of 50 μM.

Treatment of Cells *in vitro* with Caspase Inhibitors

iBMDM were passaged and seeded for LDH release cell death assay or western blot analysis as indicated above. After 24 h, cells were either left untreated, pre-treated with the caspase-1 specific inhibitor VX-765 (20 μM) or the broad-spectrum caspase inhibitor Emricasan (20 μM) for 1 h prior to infection with *Salmonella* SL1344 (MOI = 50) in the presence of inhibitor. After 1 h, cells were washed twice with warm PBS and fresh medium containing 50 μg/mL Gentamycin and the respective inhibitor added. At the indicated time points, cells were either harvested for immunoblot analysis or cell death was measured by LDH release.

Treatment of Cells *in vitro* with LPS

iBMDM) were passaged and seeded for immunoblot analysis as indicated above. After 24 h, cells were either left untreated or treated with LPS for 4 h and harvested for immunoblot analysis as described above.

Treatment of Cells in vitro with the RIPK1 Inhibitor, Nec1s, and/or TNF-α plus Birinapant and Emricasan

iBMDM) were passaged and seeded for LDH release cell death assay as indicated above. After 24 h, iBMDMs were either left untreated, pre-treated with the RIPK1 specific inhibitor, Nec1s (30 μM), for 1 h prior to infection with *Salmonella* SL1344 (MOI = 50) in the continued presence of Nec1s. After 1 h, cells were washed twice with warm PBS and fresh medium containing 50 μg/mL Gentamycin and Nec1s was added. At the indicated time points, cell death was measured by LDH release. In order to verify effective inhibition of RIPK1 by Nec1s, iBMDMs were treated with TNF-α (100 ng/mL) + Birinapant (1 μM) + Emricasan (20 μM) with or without Nec1s (30 μM) or Nec1s alone (30 μM) and harvested for LDH cell death assay at the indicated time points.

Brightfield, Confocal and Lattice Light Sheet Microscopy

For confocal and lattice light sheet microscopy, BMDMs were seeded into Nunc microscopy chamber slides at a density of 1×10^5 per well using DMEM without phenol red. After 24 h, cells were stained with MitoTracker-Deep Red FM (Thermo) at a final concentration of 50 nM for 30 min, washed twice and infected with GFP-expressing *Salmonella* SPi-2 (kind gift of Strugnell lab, Peter Doherty Institute (Hautefort et al., 2003)) as previously described. After 30 min, cells were again washed twice and fresh medium containing Gentamycin (50 μg/mL) and PI (25 μg/mL; Sigma-Aldrich) was added. Cells were imaged at the Centre for Dynamic Imaging, WEHI, using either a Zeiss LSM 980 or a custom-built Lattice light sheet system constructed as outlined in (Chen et al., 2014). Confocal images were acquired using a 1.2 NA 40x LD-LCI Plan-Apochromat lens (Zeiss) at a temperature of 37°C at 5% CO_2. For lattice light-sheet imaging, illumination at the back aperture of the excitation objective was focused through an annular mask of 0.44 inner NA and 0.55 outer NA. Fluorescent emission was collected by detection objective (Nikon, CFI Apo LWD 25XW, 1.1 NA), and detected by sCMOS cameras (Hamamatsu Orca Flash 4.0 v2). Lattice light sheet images were de-skewed and deconvolved using an iterative Richardson-Lucy algorithm before visualization. Images were processed using either Fiji or IMARIS software packages.

For Brightfield microscopy, iBMDM cells were seeded into Nunc microscopy chamber slides at a density of 1×10^5 per well using DMEM. Cells were infected with *Salmonella* SL1344 (MOI = 50) as described above and images taken on a Zeiss LSM 980. Images were processed using Fiji software package.

CRISPR/Cas9 Whole Genome Guide RNA Library Screen

293T cells were used to generate lentivirus containing the YUSA mouse full genome sgRNA library (Koike-Yusa et al., 2014). *Gsdmd*$^{-/-}$;*Bid*$^{-/-}$;*Mlkl*$^{-/-}$;*Casp3*$^{-/-}$;*Casp7*$^{-/-}$;*Casp9*$^{-/-}$ iBMDMs were seeded at a density of 1×10^{6} and lentivirally transduced with the sgRNA library. BFP+ cells were sorted using a FACSaria, expanded and seeded into 10 flasks at a density of 2×10^{6} cells. The next day, 5 flasks were infected with *Salmonella* SL1344 (MOI = 50) as described in the protocols above, and 5 flasks were harvested as non-infection control. Infected flasks were washed every 24 h for 3 days in order to remove cell debris and dying cells, and medium containing Gentamycin replaced respectively. Once surviving cells of each flask had expanded sufficiently, they were split and re-seeded at a density of 1×10^6 cells for a second round of infection. The remaining cells were frozen for analysis of guide RNA enrichment by NGS. This procedure was repeated for a third round. Genomic DNA of harvested cells from control flasks and *Salmonella*-infected flasks was extracted using the QIAGEN genomic DNA extraction kit as per the manufacturer's protocol (QIAGEN). For targeted PCR of gDNA Insertion Sites, specific primers were used to amplify from the CRISPR backbone vector which surrounds the guide RNA sequence (Aubrey et al., 2015). Individual infection and control replicates were amplified in triplicate. The PCR cycling conditions were as follows: 95°C 2 min, (95°C 15 s, 60°C 15 s, 72°C 30 s) x35 cycles, 72°C 7 min, 4°C hold step). Amplicon size distribution was ascertained using the Agilent Tapestation D1000 protocol. When a single band was observed at 250 bp, the sample was accepted as amplifying the expected target region. All reactions from the entire plate were then pooled and the PCR amplicons were bead purified as previously described. The quality and integrity of the samples was ascertained as previously described and the

concentration was used to set up the sequencing reaction. Each dual indexed library plate pool was quantified using the Agilent Tapestation and the Qubit RNA assay kit for Qubit 2.0® Fluorometer (Life Technologies). The indexed pool was diluted to 12 pM for sequencing on a MiSeq instrument as per the manufacturer's instructions. The 150-cycle kit was used to generate a single read of sequence. The fastq sequence output file from the run was used for analysis.

For analysis, the data were formatted into a matrix such that each row represented an individual guide RNA and each column a sample. Analyses of these data were then undertaken using the edgeR (Robinson et al., 2010) software package. Technical replicates were first combined using edgeR's sumTechReps function and water control samples removed prior to data filtering. Guide RNAs were filtered out if they failed to achieve a count of 10 in at least 5 samples, leaving 1384 guides for downstream analysis. Following filtering, the data were normalized using edgeR's TMM normalization (Robinson and Oshlack, 2010) with singleton pairing. For normalization only a prior count of 10 was added to all observations. This prior count was then removed for all other analyses. Differential abundance of guide RNAs between the *Salmonella*-infected and control samples was assessed using edgeR's likelihood ratio test. The false discovery rate (FDR) for this analysis was set at 5%. The mean-difference plot was generated using edgeR's plotMD function, while the heatmap was created using the pheatmap software package.

QUANTIFICATION AND STATISTICAL ANALYSIS

Prism v8.0 (GraphPad Software, San Diego, CA, USA) was used to perform statistical tests. Groups were compared by either unpaired two-tailed t tests for parametric data, or Mann–Whitney tests for non-parametric data. Survival data were analyzed using log rank (Mantel Cox) test. Please refer to the legend of the figures for description of sample size (n) and statistical significance. P values were calculated and are indicated as follows: $^{**}p < 0.005$; $^{*}p < 0.05$; $^{ns}p > 0.05$ = not significant (ns).

Immunity, Volume *53*

Supplemental Information

Flexible Usage and Interconnectivity of Diverse Cell Death Pathways Protect against Intracellular Infection

Marcel Doerflinger, Yexuan Deng, Paul Whitney, Ranja Salvamoser, Sven Engel, Andrew J. Kueh, Lin Tai, Annabell Bachem, Elise Gressier, Niall D. Geoghegan, Stephen Wilcox, Kelly L. Rogers, Alexandra L. Garnham, Michael A. Dengler, Stefanie M. Bader, Gregor Ebert, Jaclyn S. Pearson, Dominic De Nardo, Nancy Wang, Chenying Yang, Milton Pereira, Clare E. Bryant, Richard A. Strugnell, James E. Vince, Marc Pellegrini, Andreas Strasser, Sammy Bedoui, and Marco J. Herold

SUPPLEMENTAL INFORMATION

Supplementary Figures

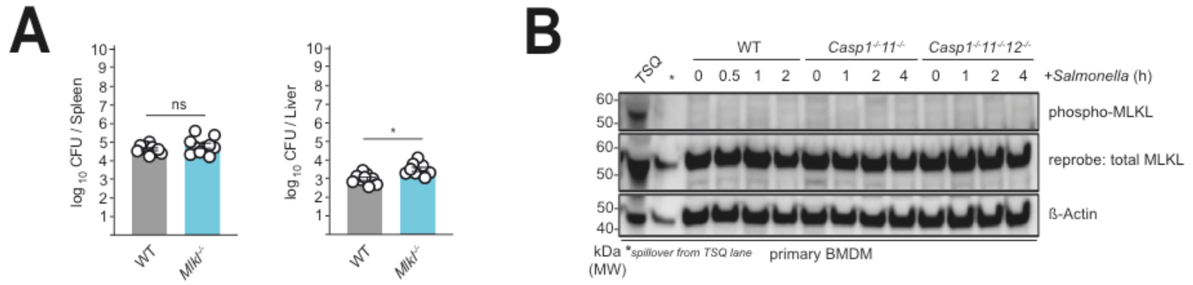

Figure S1 (related to Figure 1): Necroptosis Does not Affect Bacterial Clearance or Provide Back-up for Loss of Pyroptosis

(A) Bacterial loads in spleen and liver 3-week post infection in WT and *Mlkl*$^{-/-}$ mice infected with *Salmonella ΔAroA* (200 CFU). *N*=8 mice per group. Mean and SEM are shown. *p<0.05, ^{ns}p>0.05=not significant.

(B) WT, *Casp1*$^{-/-}$*;Casp11*$^{-/-}$ and *Casp1*$^{-/-}$*;Casp11*$^{-/-}$*;Casp12*$^{-/-}$ BMDMs were infected with *Salmonella* SL1344 (MOI=50) and phosphorylation of MLKL analyzed by Western blotting at the indicated time points. Probing for ß-actin served as a loading control. Treatment with TNF-α+ Smac mimetic + caspase inhibitor QVD-OPH (TSQ) was used as a positive control for activation (i.e. phosphorylation) of MLKL and induction of necroptosis.

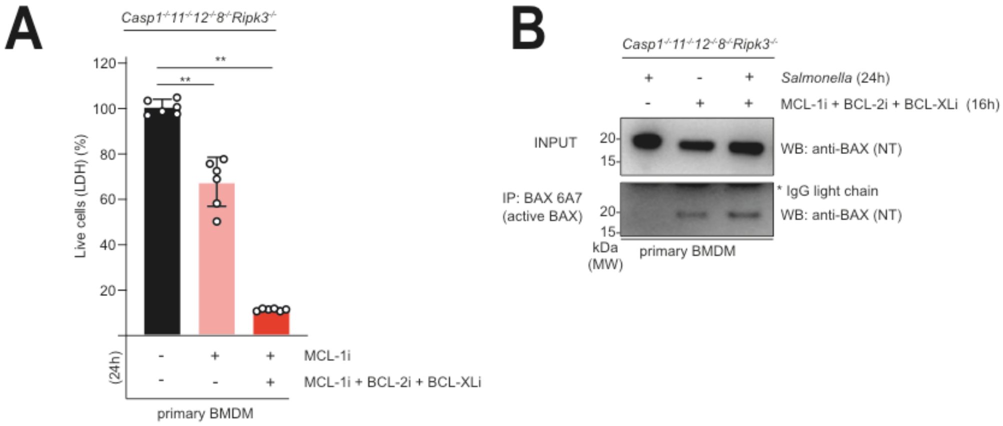

Figure S2 (related to Figure 2). Primary BMDMs from *Casp1*$^{-/-}$*;Casp11*$^{-/-}$*;Casp12*$^{-/-}$*;Casp8*$^{-/-}$*;Ripk3*$^{-/-}$ Mice Are Susceptible to Intrinsic Apoptosis Induced by BH3 Mimetic Drugs.

(A) *Casp1*$^{-/-}$*;Casp11*$^{-/-}$*;Casp12*$^{-/-}$*;Casp8*$^{-/-}$*;Ripk3*$^{-/-}$ BMDMs were treated with BH3 mimetic drugs individually (2 µM MCL1i) or in combination (each 2 µM of MCL-1i + BCL-2i + BCL-XLi) as indicated. Cell death was measured using an LDH release assay. Data pooled from 2 or more experiments. Mean and SD are shown. **P<0.005.

(B) Immunoprecipitation and immunoblotting of activated BAX in *Casp1*$^{-/-}$*;Casp11*$^{-/-}$*;Casp12*$^{-/-}$*;Casp8*$^{-/-}$*;Ripk3*$^{-/-}$ BMDMs infected with *Salmonella* SL1344 (MOI=50) and/or treated with the BH3 mimetic drug combination (each 2 µM of MCL-1i + BCL-2i + BCL-XLi).

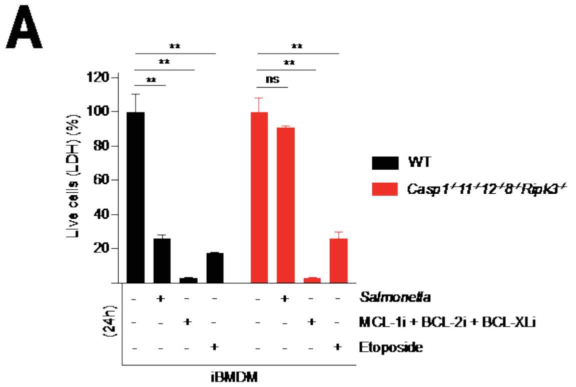

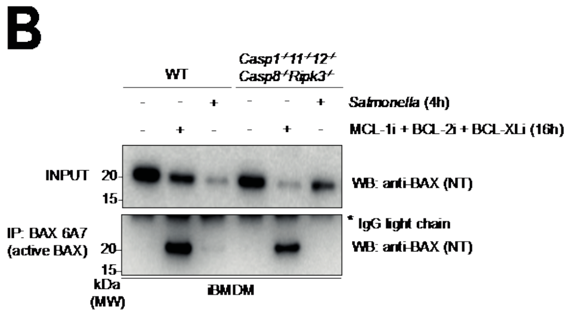

Figure S3 (related to Figure 3). ***Casp1*$^{-/-}$*;Casp11*$^{-/-}$*;Casp12*$^{-/-}$*;Casp8*$^{-/-}$*;Ripk3*$^{-/-}$ iBMDMs Undergo Intrinsic Apoptosis in Response to BH3 Mimetic Drugs or Etoposide**

(A) LDH release cell death assay of WT and *Casp1*$^{-/-}$*;Casp11*$^{-/-}$*;Casp12*$^{-/-}$*;Casp8*$^{-/-}$*;Ripk3*$^{-/-}$ iBMDMs after 24 h treatment with a combination of BH3 mimetic drugs (each 2 µM of MCL-1i + BCL-2i + BCL-XLi), Etoposide (50 µM) or infection with *Salmonella* SL1344 (MOI=50). Mean and SEM are shown. **p<0.005; ^{ns}p>0.05=not significant.

(B) Immunoprecipitation and immunoblotting of activated BAX in WT and *Casp1*$^{-/-}$*;Casp11*$^{-/-}$*;Casp12*$^{-/-}$*;Casp8*$^{-/-}$*;Ripk3*$^{-/-}$ iBMDMs infected with *Salmonella* SL1344 (MOI=50) or treated with a BH3 mimetic drug combination (each 2 µM of MCL-1i + BCL-2i + BCL-XLi).

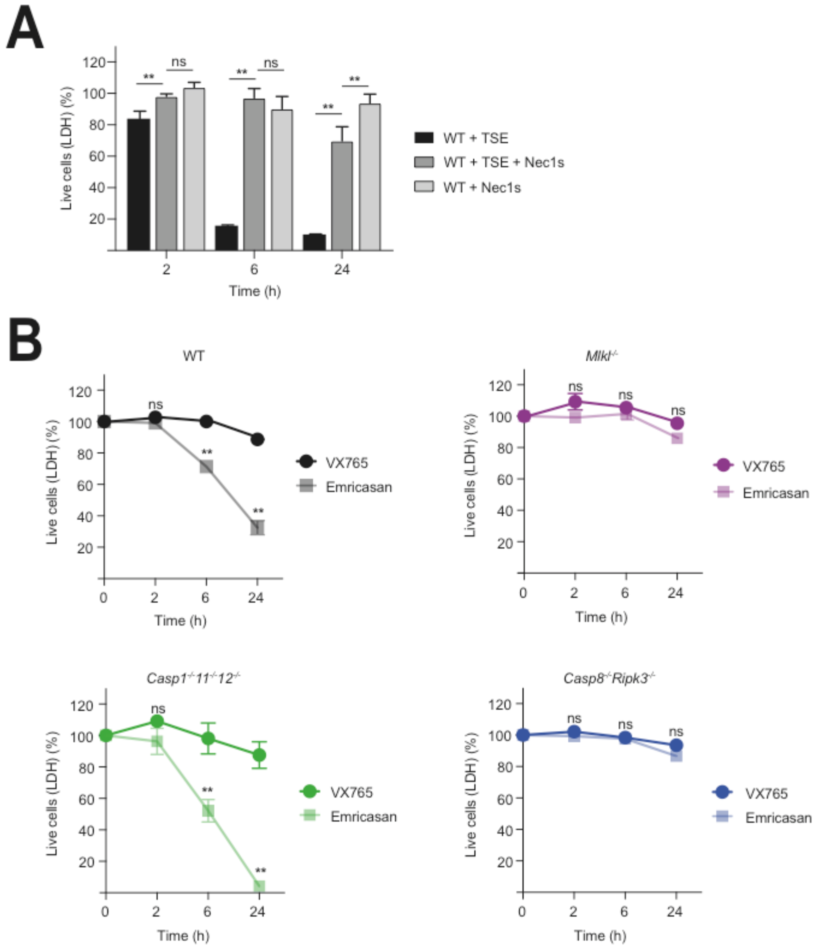

Figure S4 (related to Figures 3 and 4): Impact of RIPK1 Inhibition by Nec1s and Effect of Caspase-1 Inhibition or Inhibition of All Caspases on the Survival of iBMDM.

(A) WT iBMDMs were treated with TNF-α (100 ng/mL) + Birinapant (1 μM) + Emricasan (20 μM) with or without the RIPK1 inhibitor Nec1s (30 μM) and cell death was measured by LDH release. Data pooled from 2 or more experiments. Mean and SEM are shown. **$p<0.005$; $^{ns}p>0.05$=not significant.

(B) LDH release cell death assays of WT, *MLKL*$^{-/-}$, *Casp1*$^{-/-}$;*Casp11*$^{-/-}$;*Casp12*$^{-/-}$, *Casp8*$^{-/-}$;*Ripk3*$^{-/-}$ and *Casp1*$^{-/-}$;*Casp11*$^{-/-}$;*Casp12*$^{-/-}$;*Casp8*$^{-/-}$;*Ripk3*$^{-/-}$ iBMDMs that had been treated with the caspase-1 specific inhibitor, VX-765 (20 μM) or the broad-spectrum caspase inhibitor, Emricasan (20 μM). Data pooled from 2 or more experiments. Mean and SEM are shown. **$p<0.005$; $^{ns}p>0.05$=not significant.

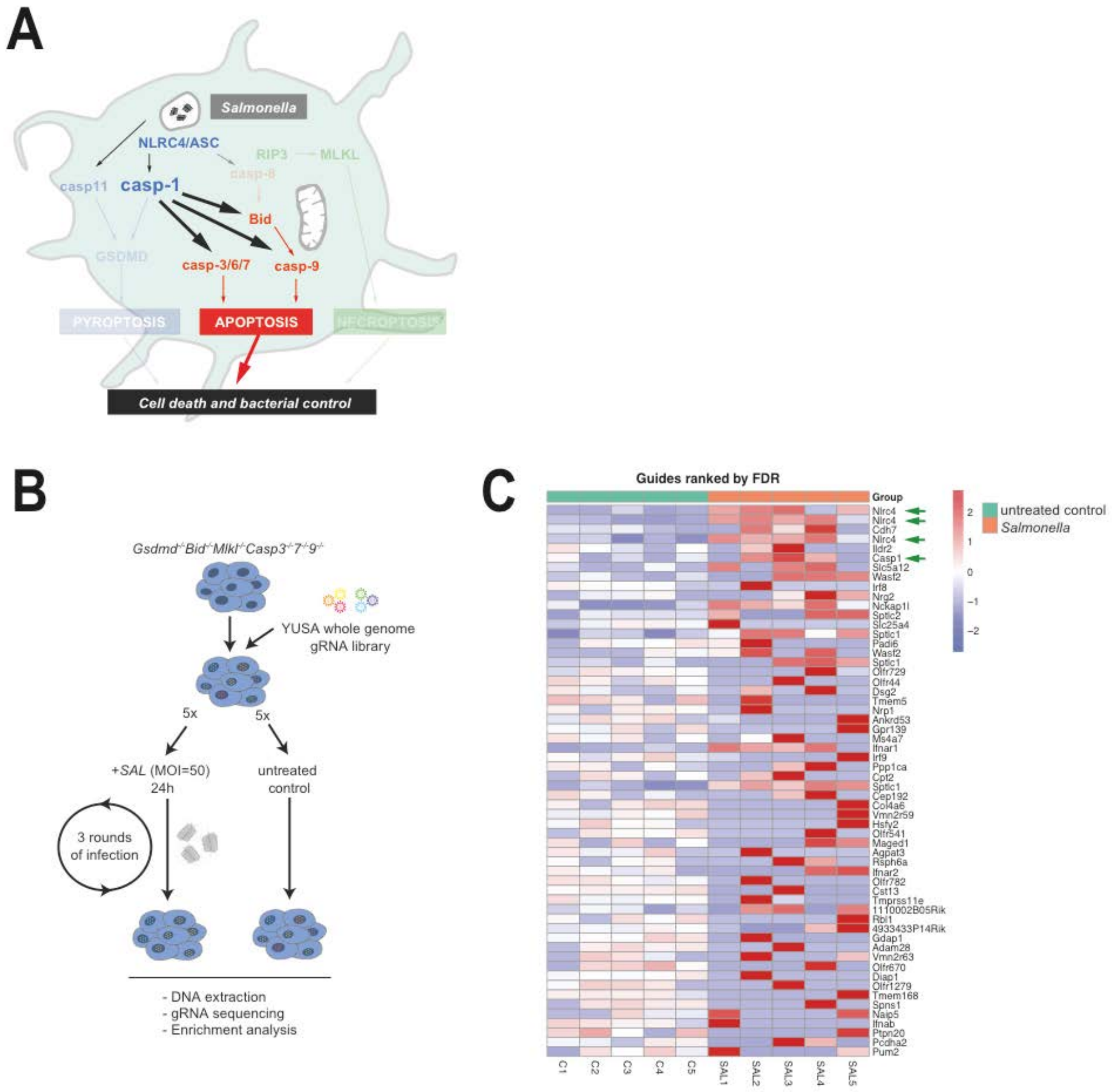

Figure S5 (related to Figures 5 and 6). CRISPR/Cas9 Screen Identifies Caspase-1 and NRLC4 as the Central Drivers of Cell Death in the Absence of all known Downstream Effectors of Cell Killing.

(A) Schematic overview of cell death induction by caspase-1 in the absence of pyroptosis and caspase-8.

(B) Schematic overview of CRISPR/Cas9 whole genome screen: *GsdmD*$^{-/-}$;*Bid*$^{-/-}$;*Mlkl*$^{-/-}$;*Casp3*$^{-/-}$;*Casp7*$^{-/-}$;*Casp9*$^{-/-}$ iBMDMs were transduced with a whole genome sgRNA library (Koike-Yusa et al., 2014) and infected in replicates for three consecutive rounds with *Salmonella* SL1344 (MOI=50). Surviving cells were expanded and subjected to NGS analysis.

(C) A heatmap of the sgRNAs significantly enriched during the CRISPR/Cas9 screen as shown per individual replicate, ranked according to false discovery rate. Green arrowheads indicate sgRNAs for caspase-1 and NLRC4.

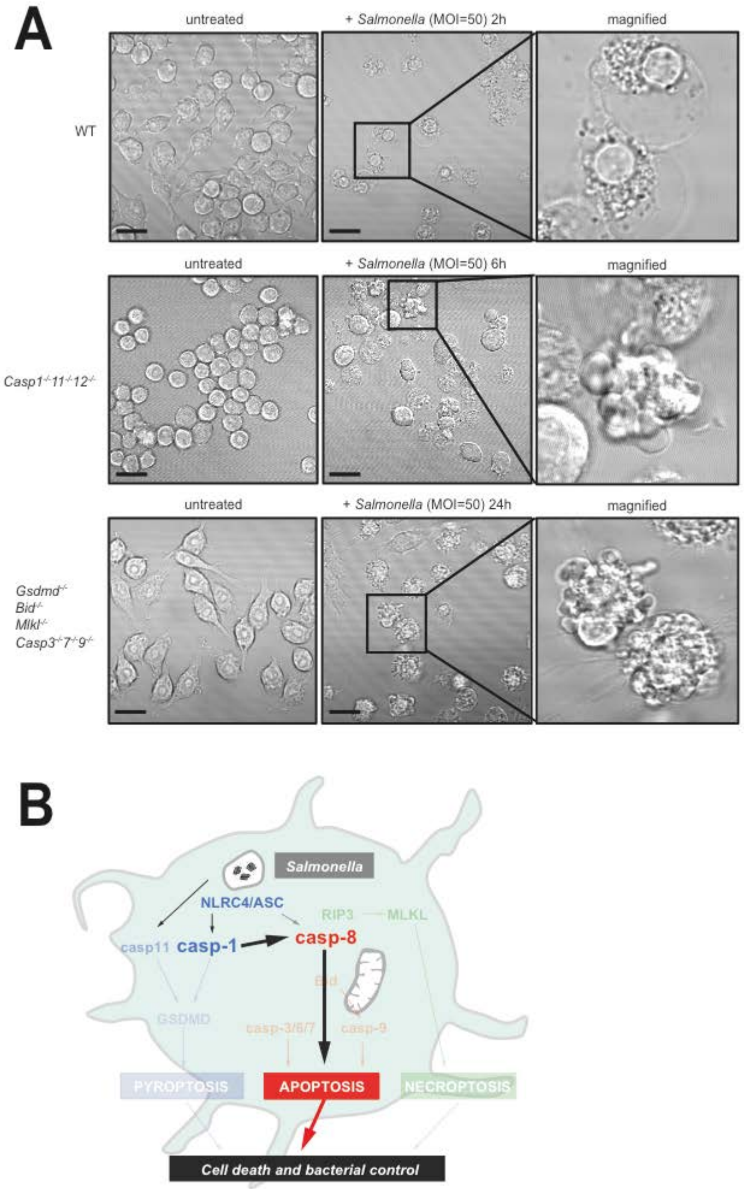

Figure S6 (related to Figures 6 and 7). Macrophages Lacking All Downstream Effectors of Caspase-1 and Caspase-8 Undergo Delayed Apoptotic Cell Death upon *Salmonella* Infection.

(A) WT, *Casp1*$^{-/-}$;*Casp11*$^{-/-}$;*Casp12*$^{-/-}$and *GsdmD*$^{-/-}$;*Bid*$^{-/-}$;*Mlkl*$^{-/-}$;*Casp3*$^{-/-}$; *Casp 7*$^{-/-}$; *Casp 9*$^{-/-}$ iBMDM cells were left untreated or infected with *Salmonella* SL1344 (MOI=50) and analyzed using brightfield microscopy. Scale bar: 20 µm.

(B) Schematic of cell death pathway hierarchy in the absence of all known downstream effectors of apoptosis, pyroptosis and necroptosis
